# Supplementary material for: Firearm Safety Counseling for Patients: An Interactive Curriculum for Trauma Providers
Source: MedEdPORTAL. 2022 May 10;18:11237. doi: 10.15766/mep_2374-8265.11237 (PMC9085984; doi:10.15766/mep_2374-8265.11237)
Supplement: Supplementary file 1 — Safe Firearm Storage.pptxStandardized Patient Cases.docxPresentation of Standardized Patient Cases.docxPre- and Postsurveys.docx [file mep_2374-8265.11237-s001.zip › A. Safe Firearm Storage.pptx]

## Slide 1
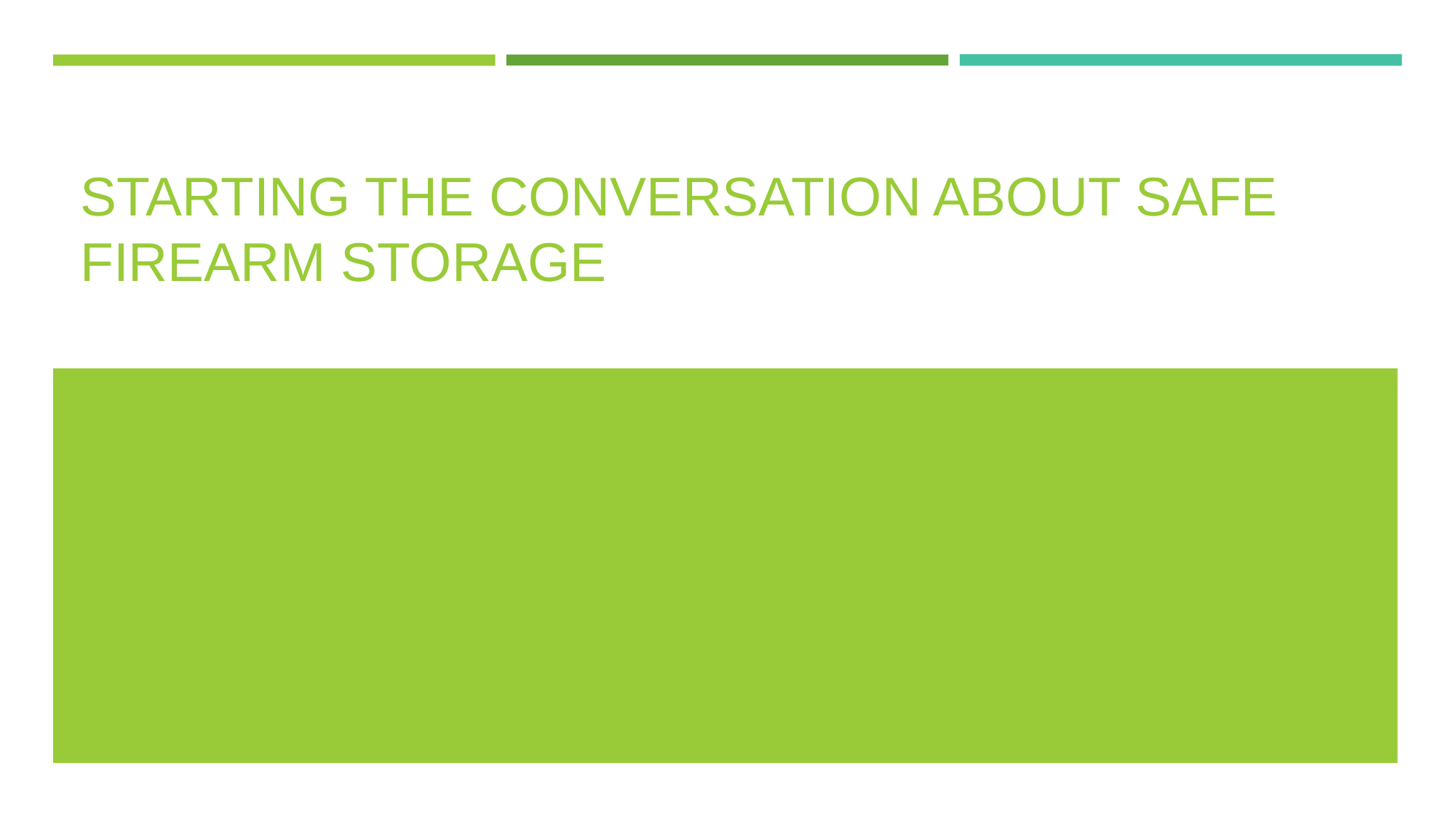

# STARTING THE CONVERSATION ABOUT SAFE FIREARM STORAGE

## Slide 2
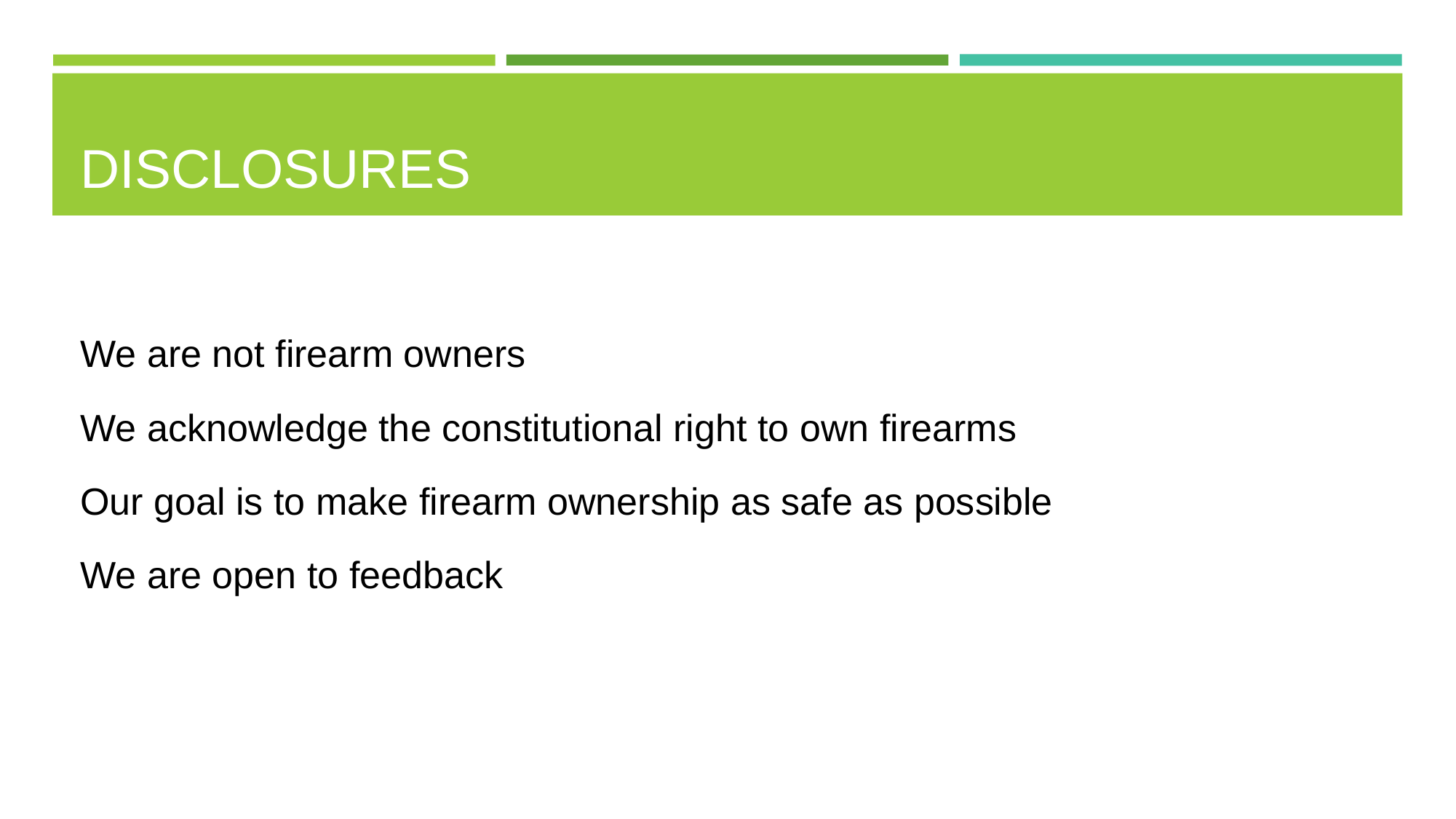

# DISCLOSURES
We are not firearm owners
We acknowledge the constitutional right to own firearms
Our goal is to make firearm ownership as safe as possible
We are open to feedback

## Slide 3
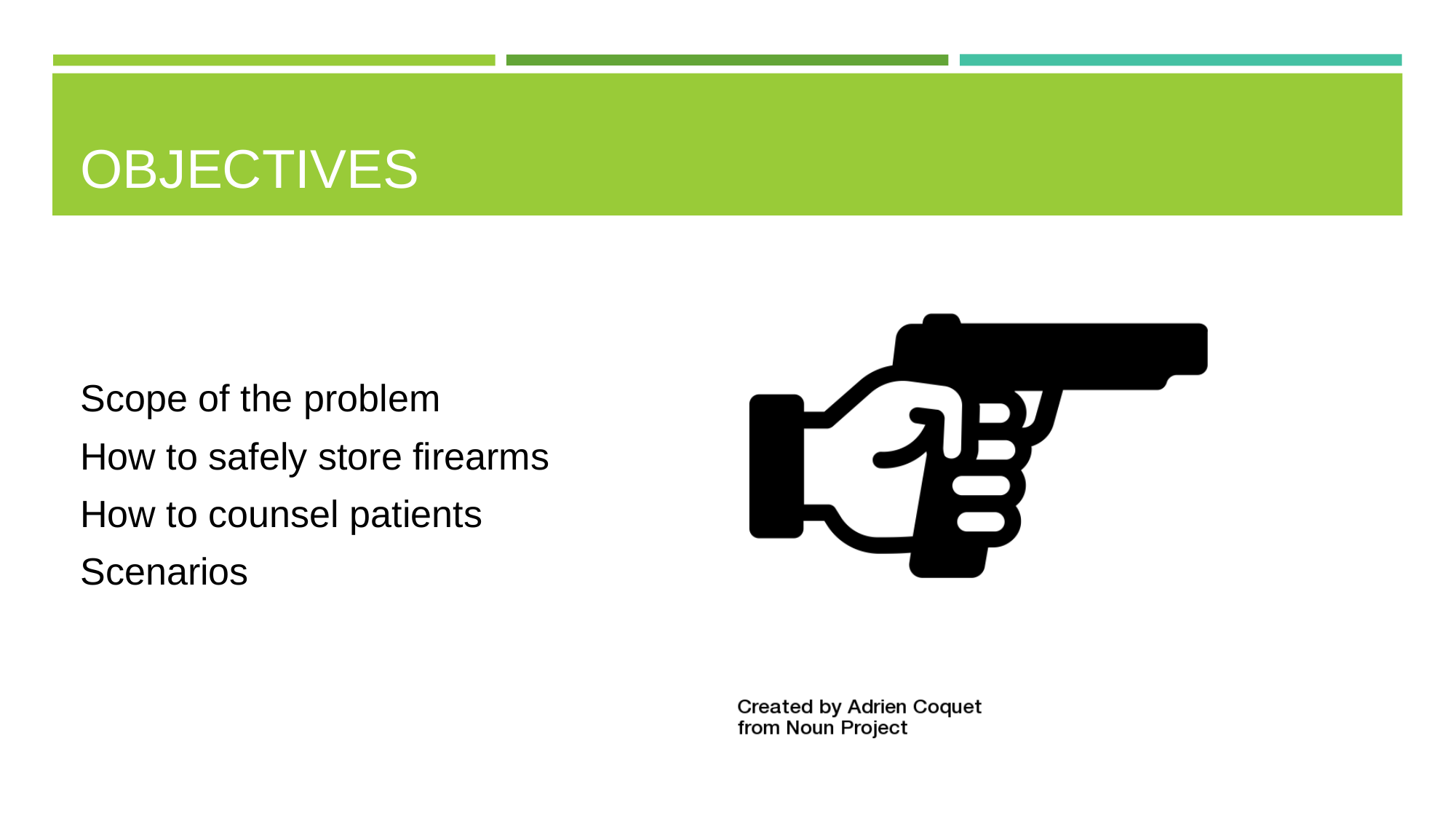

# OBJECTIVES
Scope of the problem
How to safely store firearms
How to counsel patients
Scenarios

## Slide 4
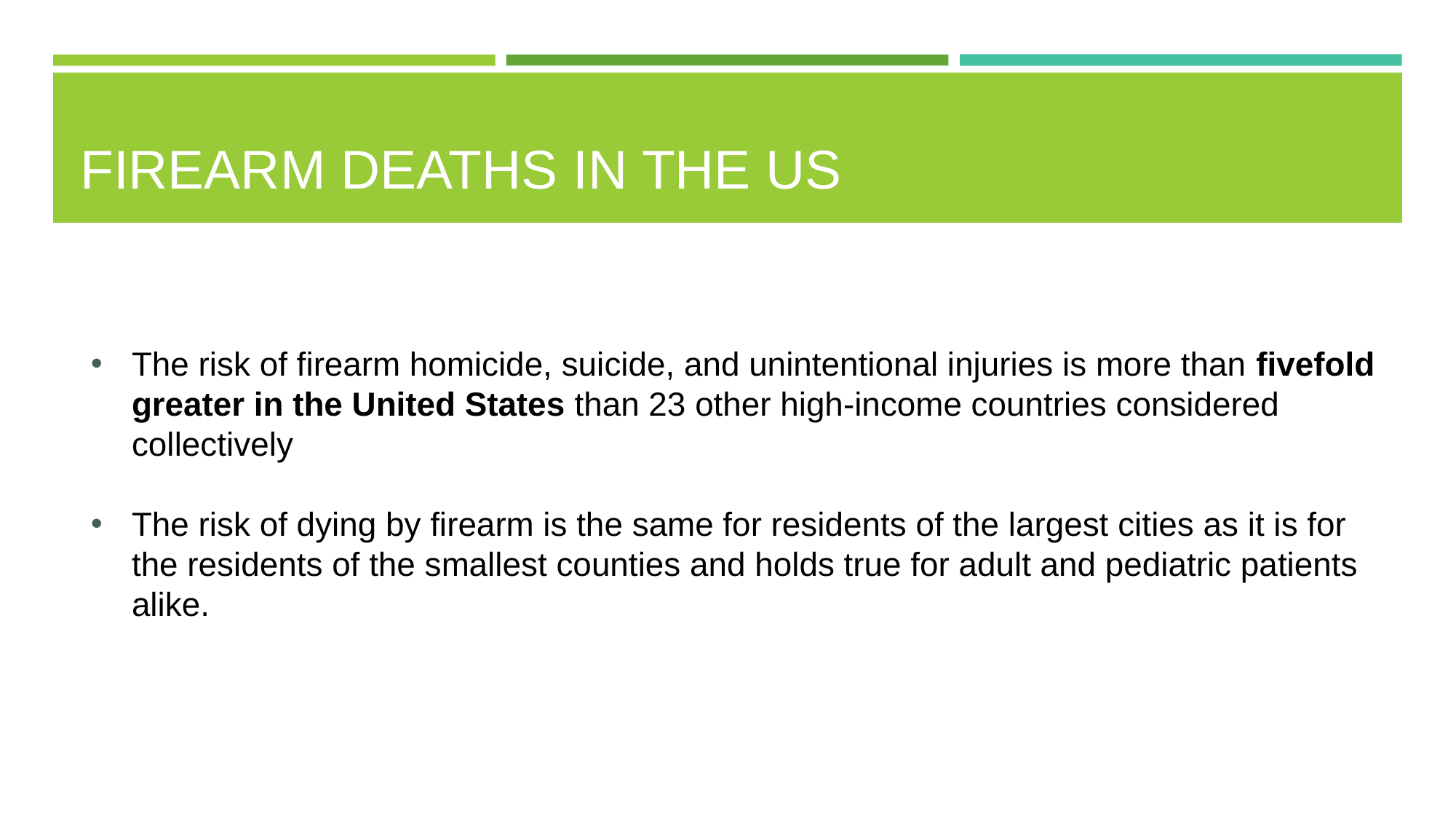

# FIREARM DEATHS IN THE US
The risk of firearm homicide, suicide, and unintentional injuries is more than fivefold greater in the United States than 23 other high-income countries considered collectively
The risk of dying by firearm is the same for residents of the largest cities as it is for the residents of the smallest counties and holds true for adult and pediatric patients alike.

## Slide 5
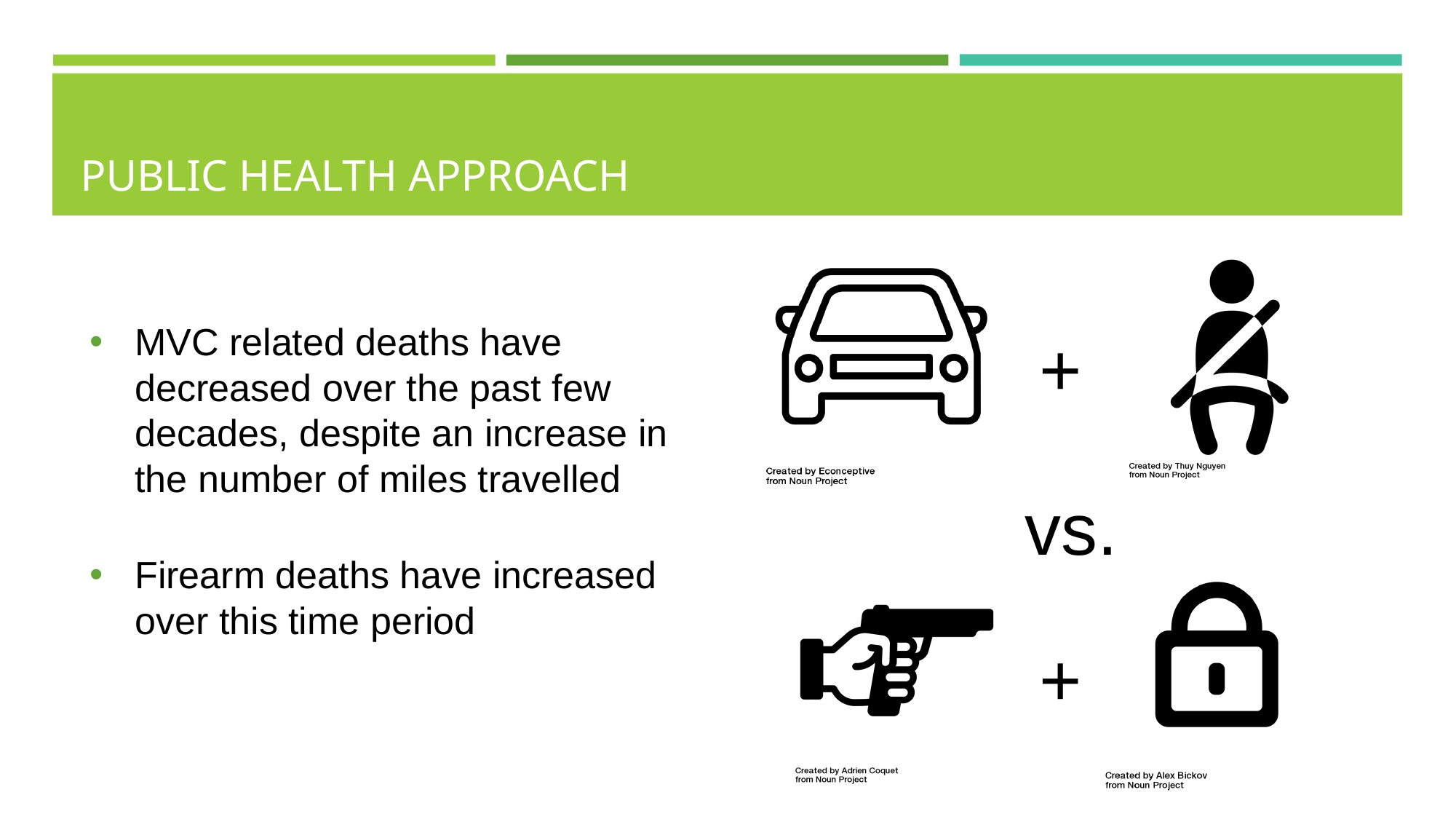

# PUBLIC HEALTH APPROACH
MVC related deaths have decreased over the past few decades, despite an increase in the number of miles travelled
Firearm deaths have increased over this time period
+
vs.
+

## Slide 6
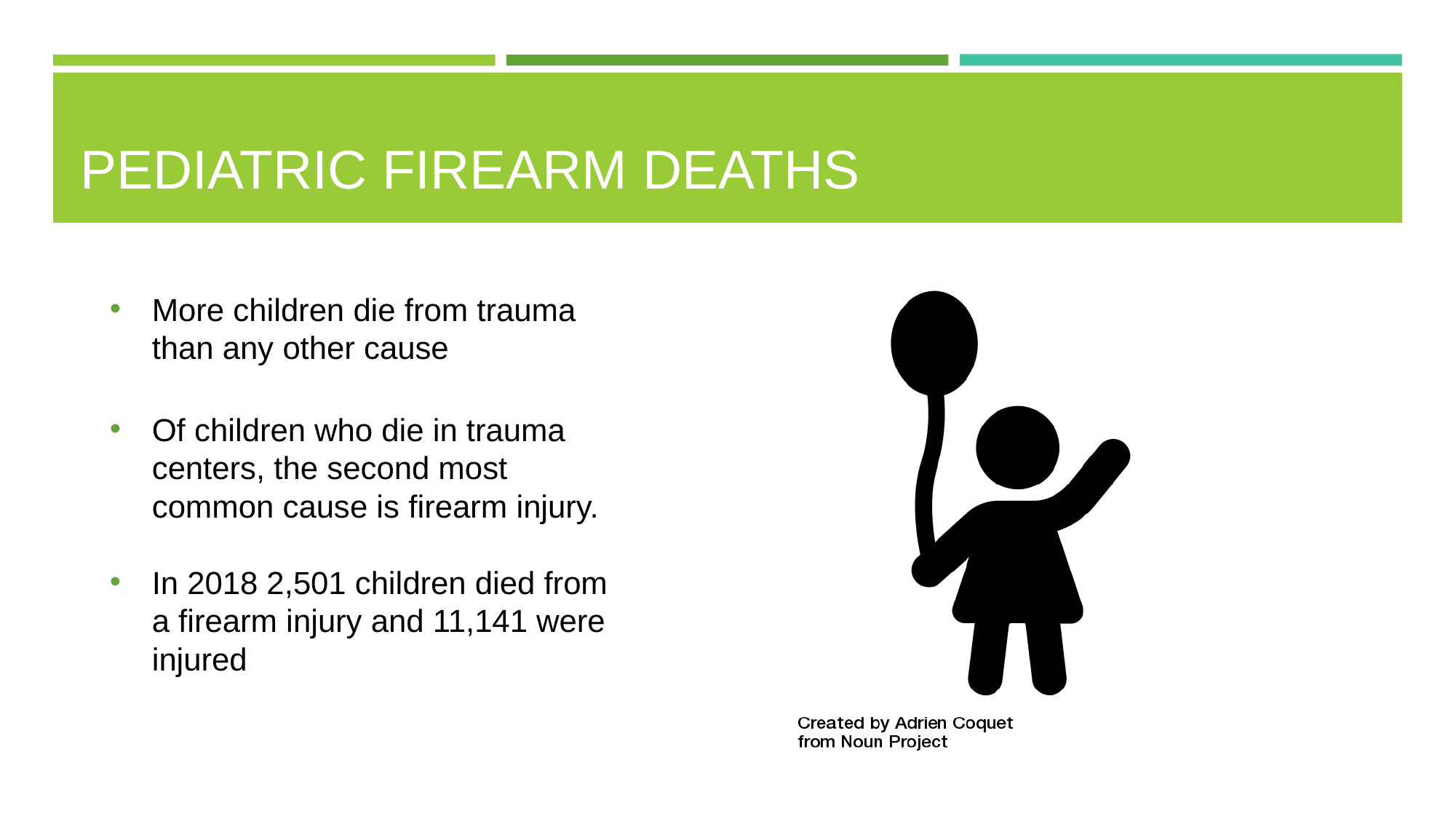

# PEDIATRIC FIREARM DEATHS
More children die from trauma than any other cause
Of children who die in trauma centers, the second most common cause is firearm injury.
In 2018 2,501 children died from a firearm injury and 11,141 were injured

## Slide 7
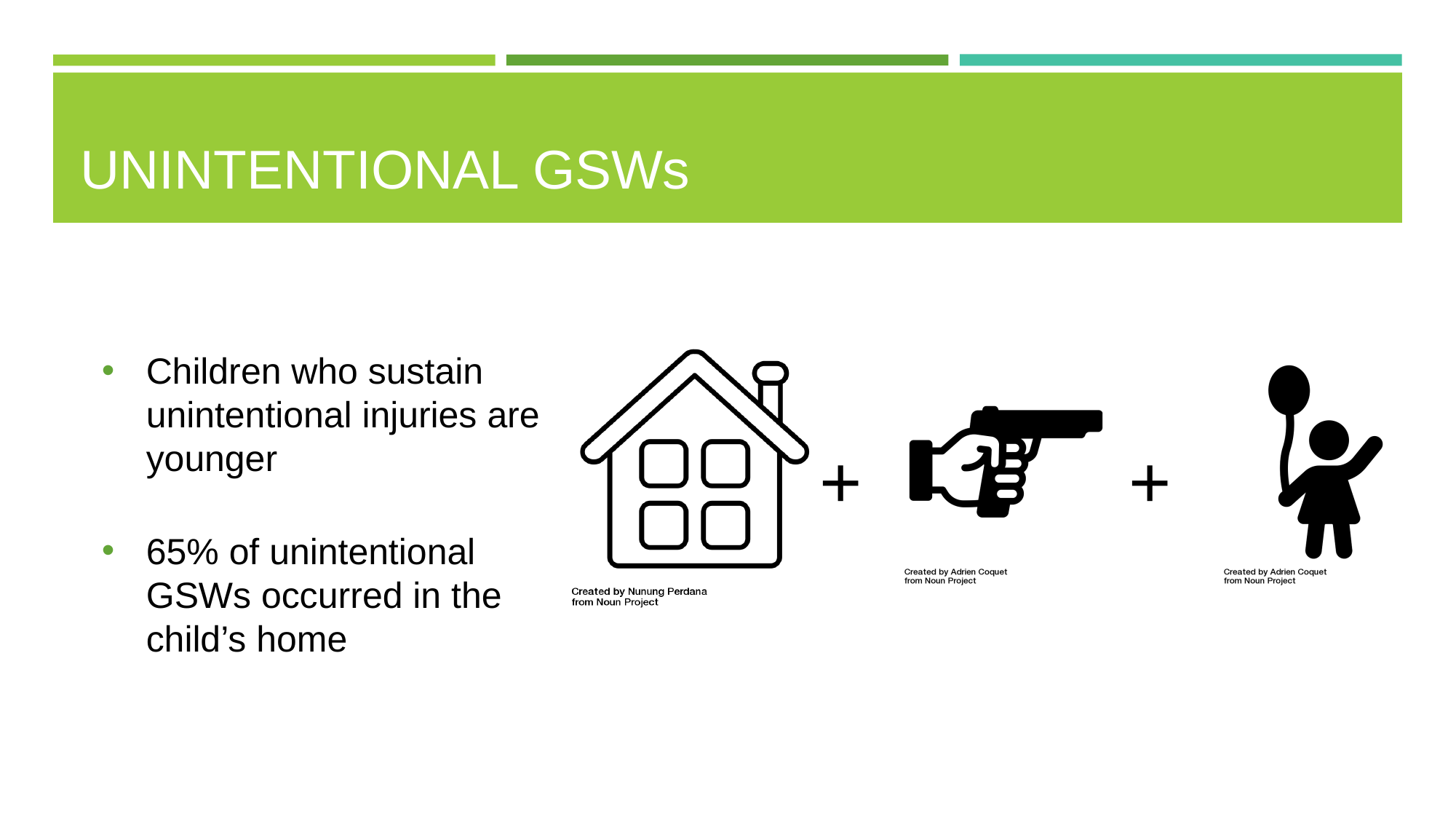

# UNINTENTIONAL GSWs
Children who sustain unintentional injuries are younger
65% of unintentional GSWs occurred in the child’s home
+
+

## Slide 8
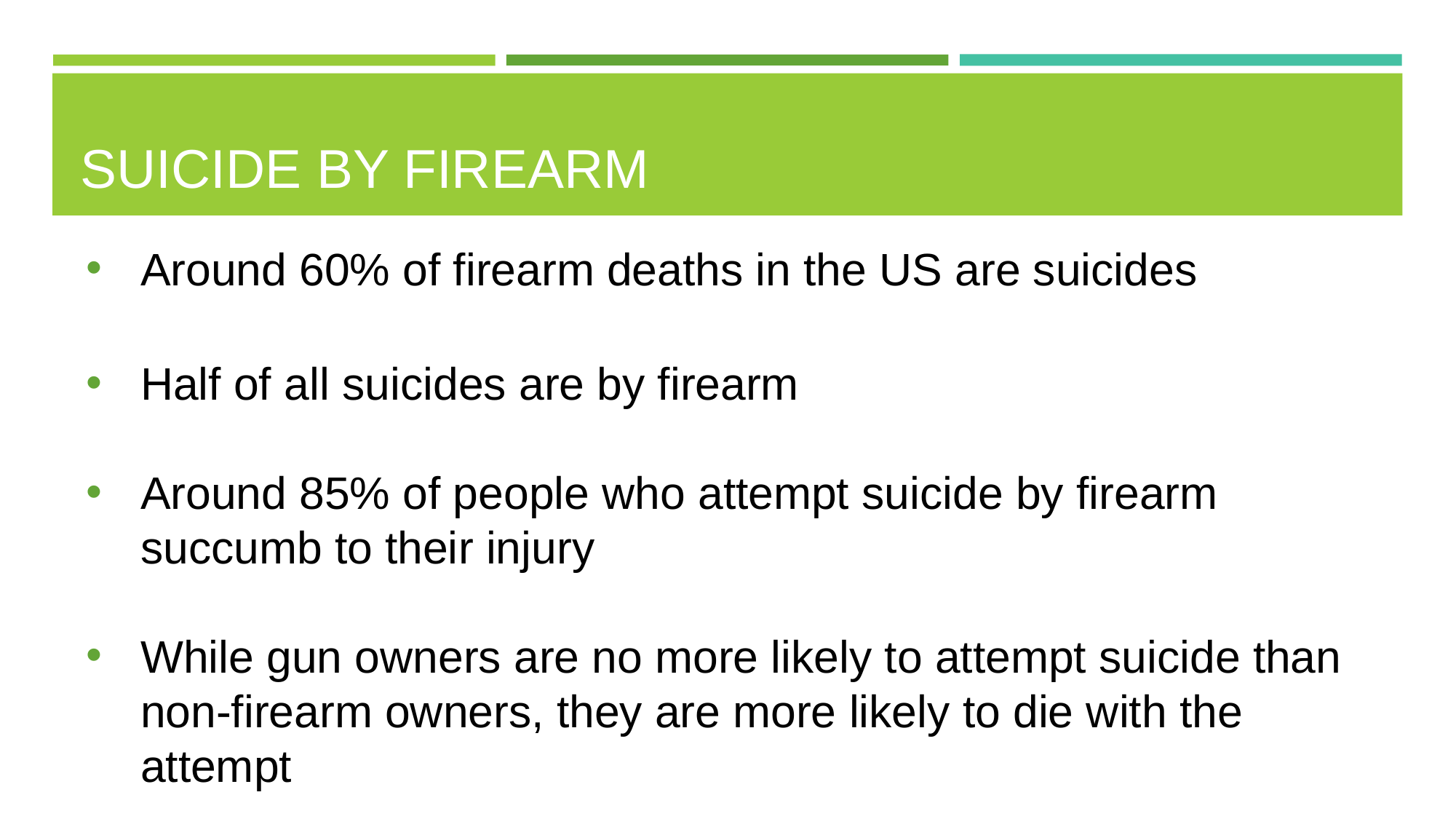

# SUICIDE BY FIREARM
Around 60% of firearm deaths in the US are suicides
Half of all suicides are by firearm
Around 85% of people who attempt suicide by firearm succumb to their injury
While gun owners are no more likely to attempt suicide than non-firearm owners, they are more likely to die with the attempt

## Slide 9
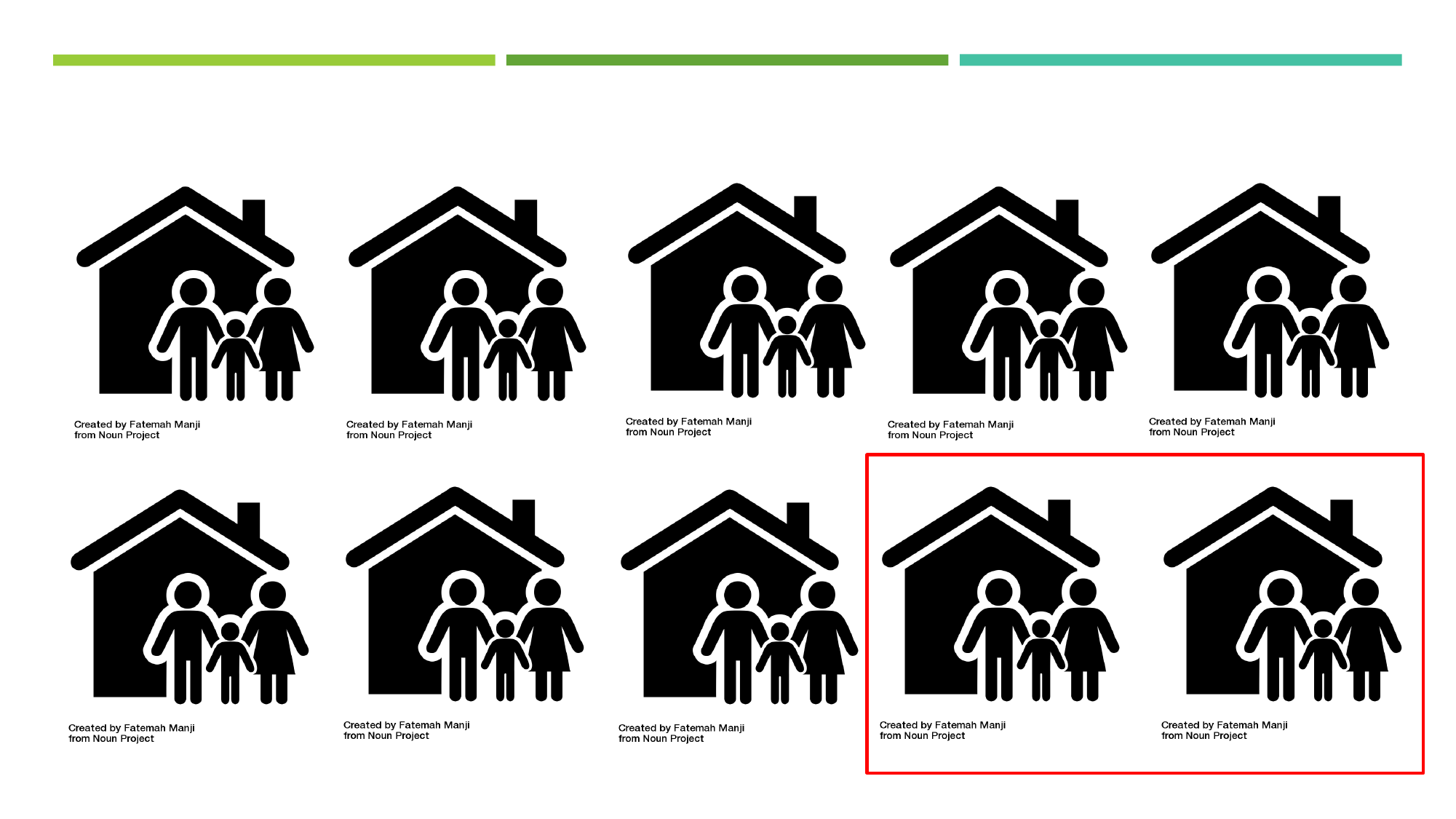

## Slide 10
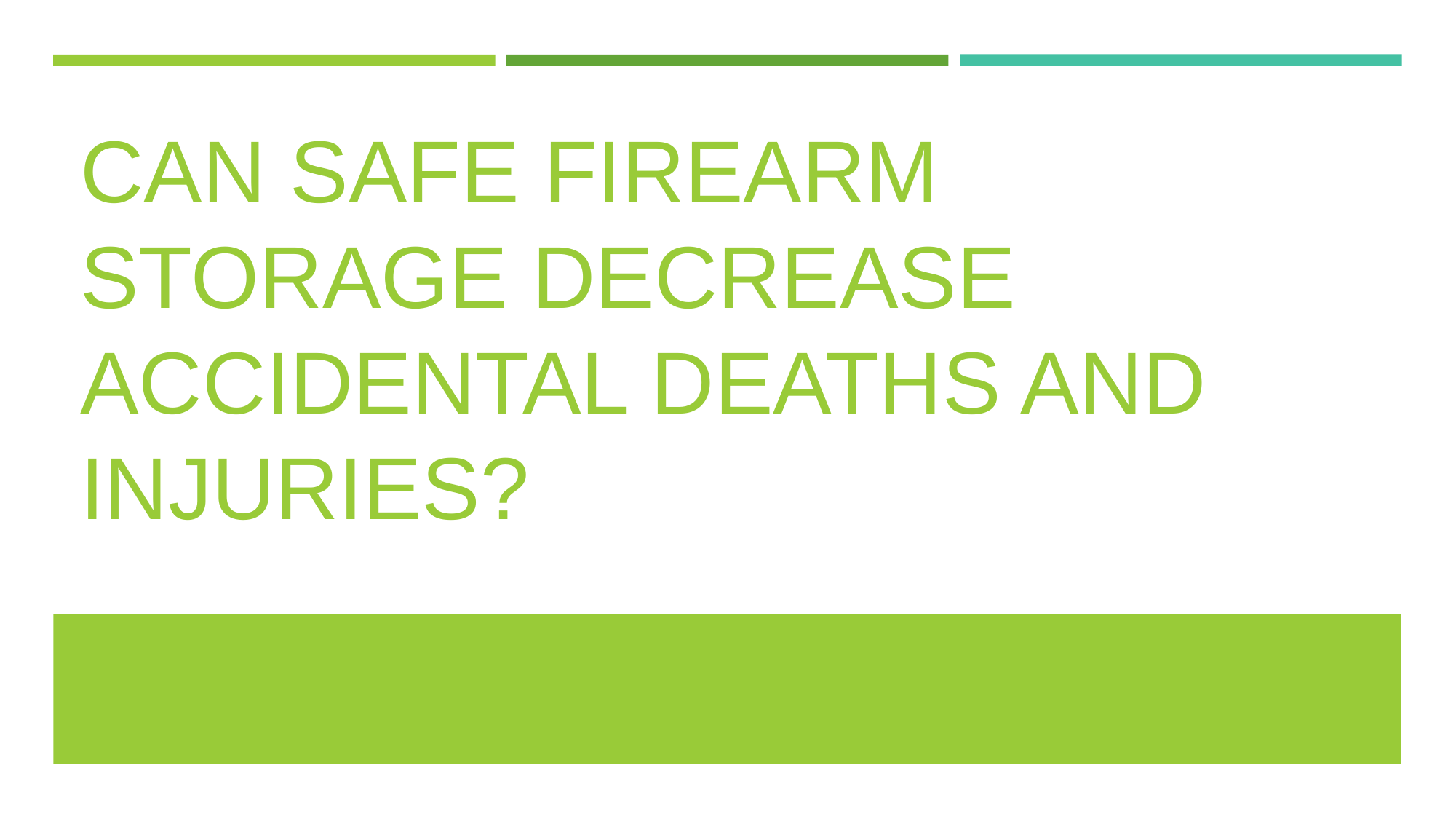

# CAN SAFE FIREARM STORAGE DECREASE ACCIDENTAL DEATHS AND INJURIES?

## Slide 11
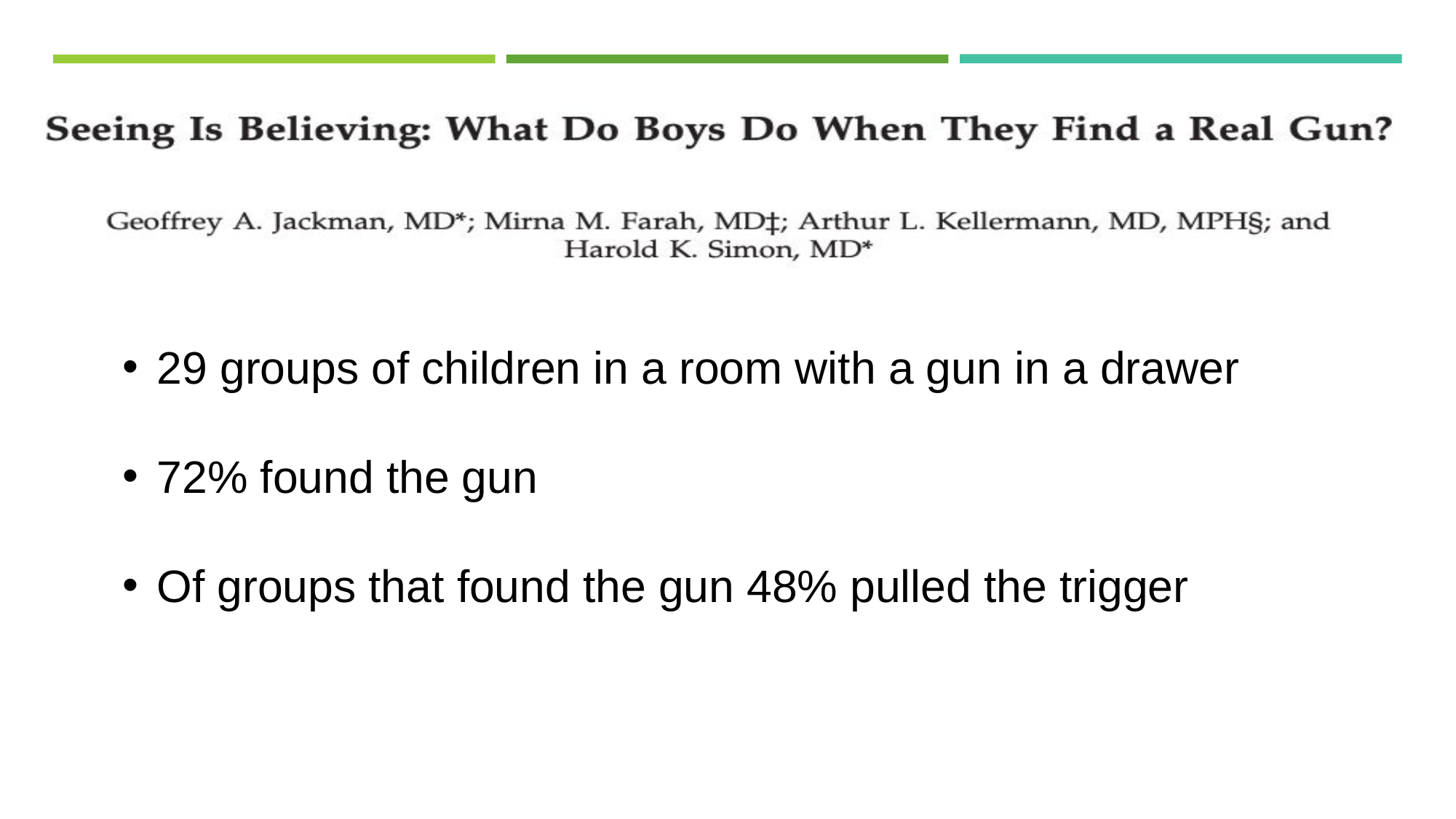

#
29 groups of children in a room with a gun in a drawer
72% found the gun
Of groups that found the gun 48% pulled the trigger

## Slide 12
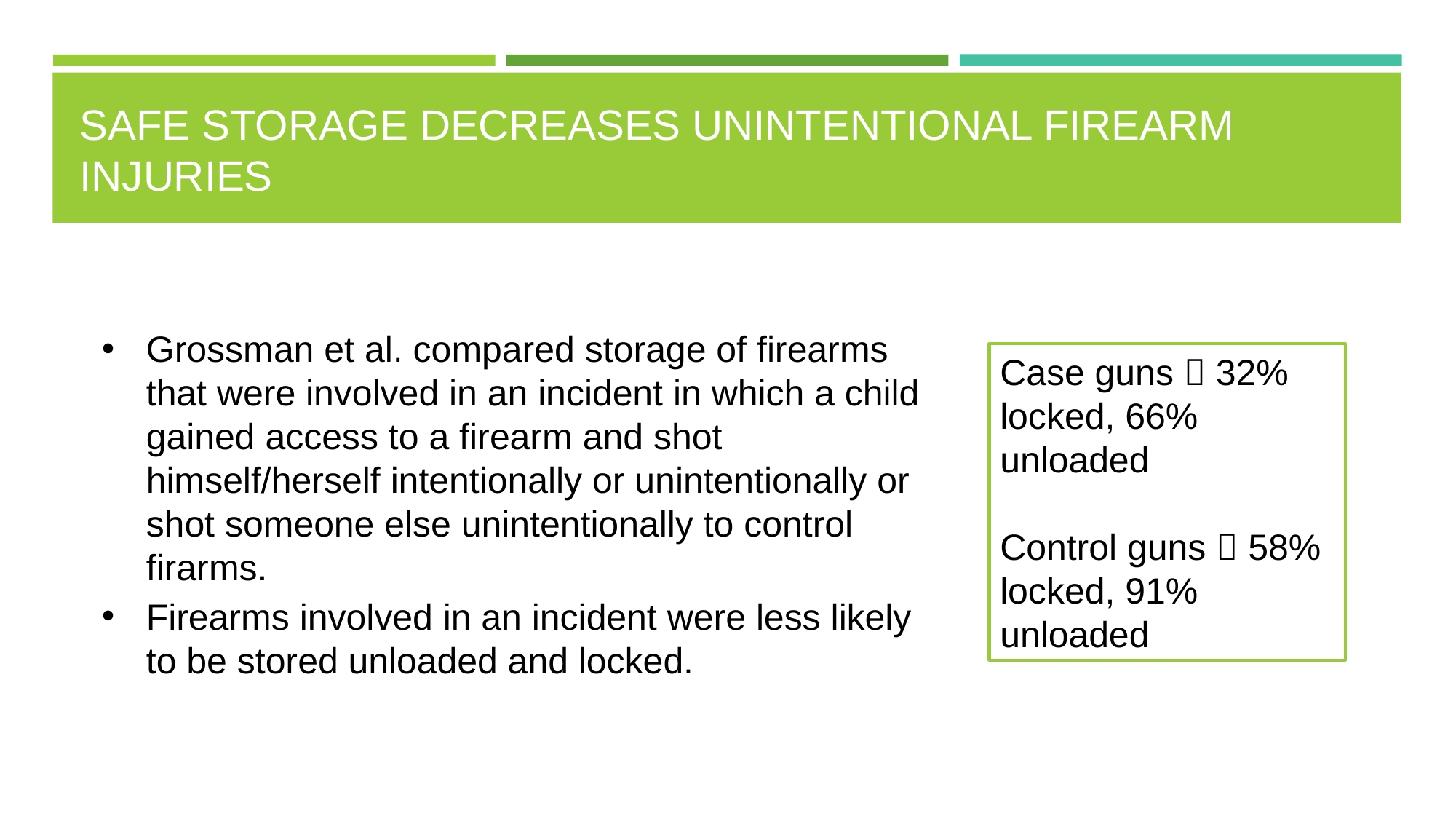

# SAFE STORAGE DECREASES UNINTENTIONAL FIREARM INJURIES
Grossman et al. compared storage of firearms that were involved in an incident in which a child gained access to a firearm and shot himself/herself intentionally or unintentionally or shot someone else unintentionally to control firarms.
Firearms involved in an incident were less likely to be stored unloaded and locked.
Case guns  32% locked, 66% unloaded
Control guns  58% locked, 91% unloaded

## Slide 13
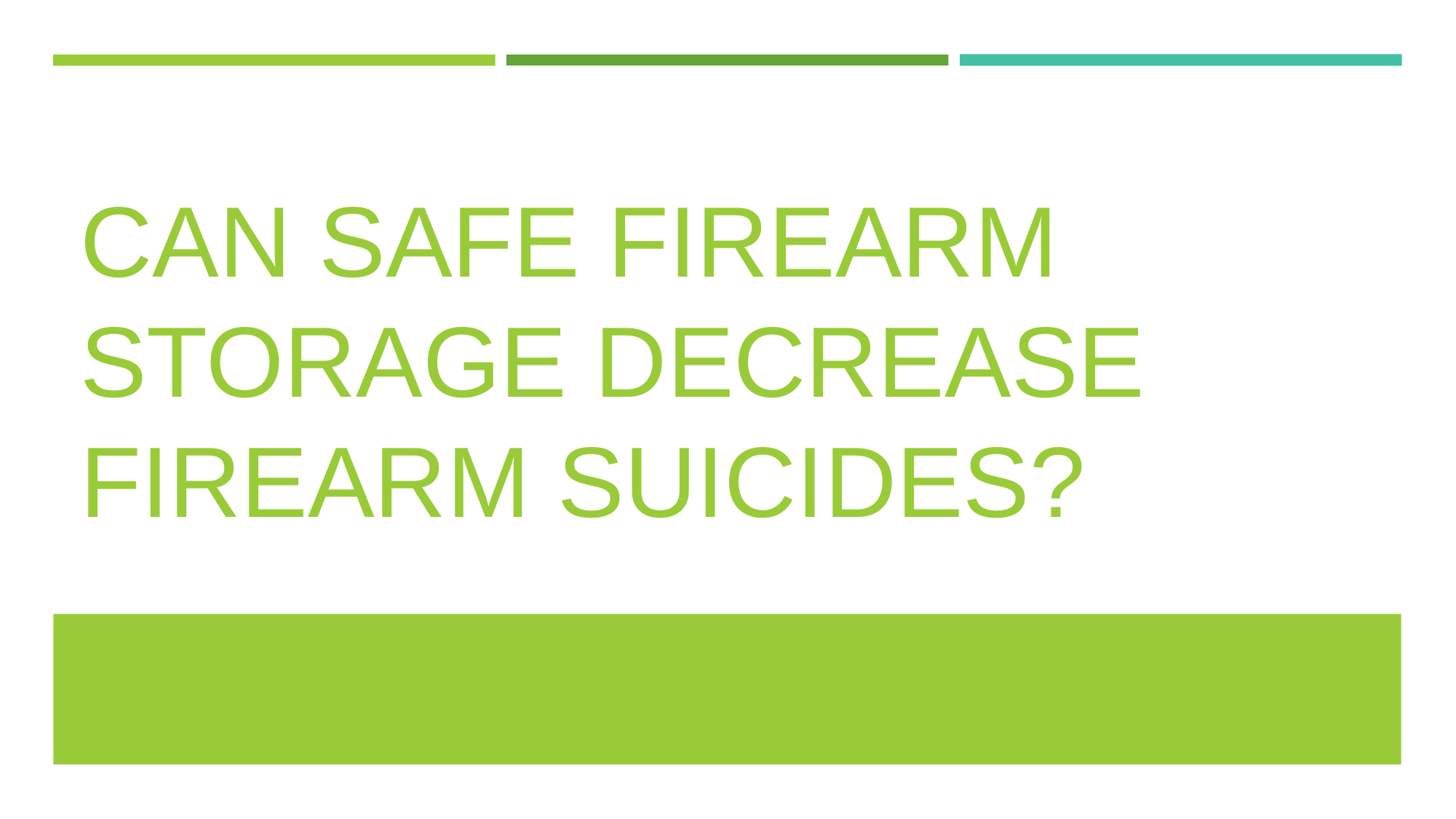

# CAN SAFE FIREARM STORAGE DECREASE FIREARM SUICIDES?

## Slide 14
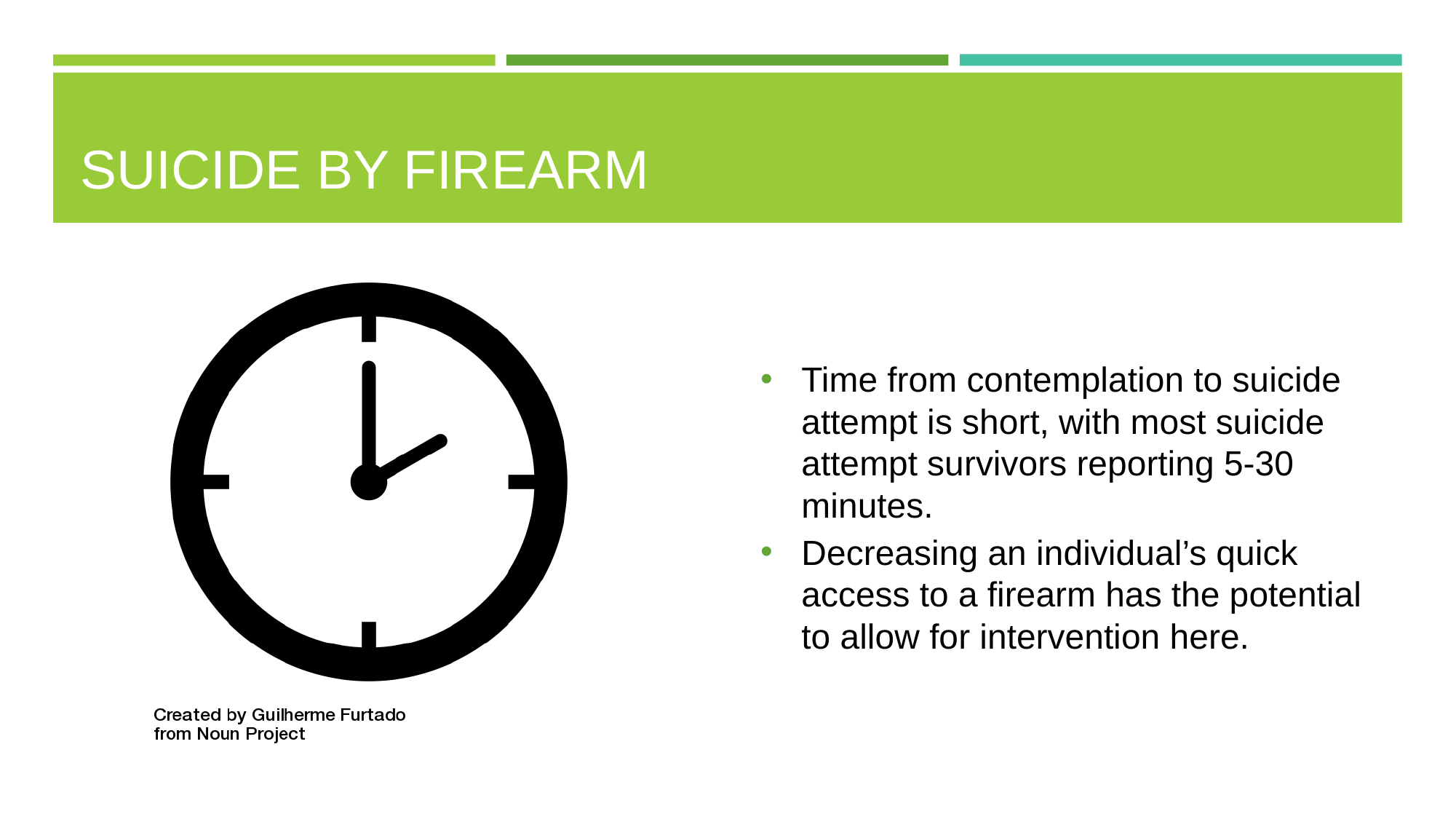

# SUICIDE BY FIREARM
Time from contemplation to suicide attempt is short, with most suicide attempt survivors reporting 5-30 minutes.
Decreasing an individual’s quick access to a firearm has the potential to allow for intervention here.

## Slide 15
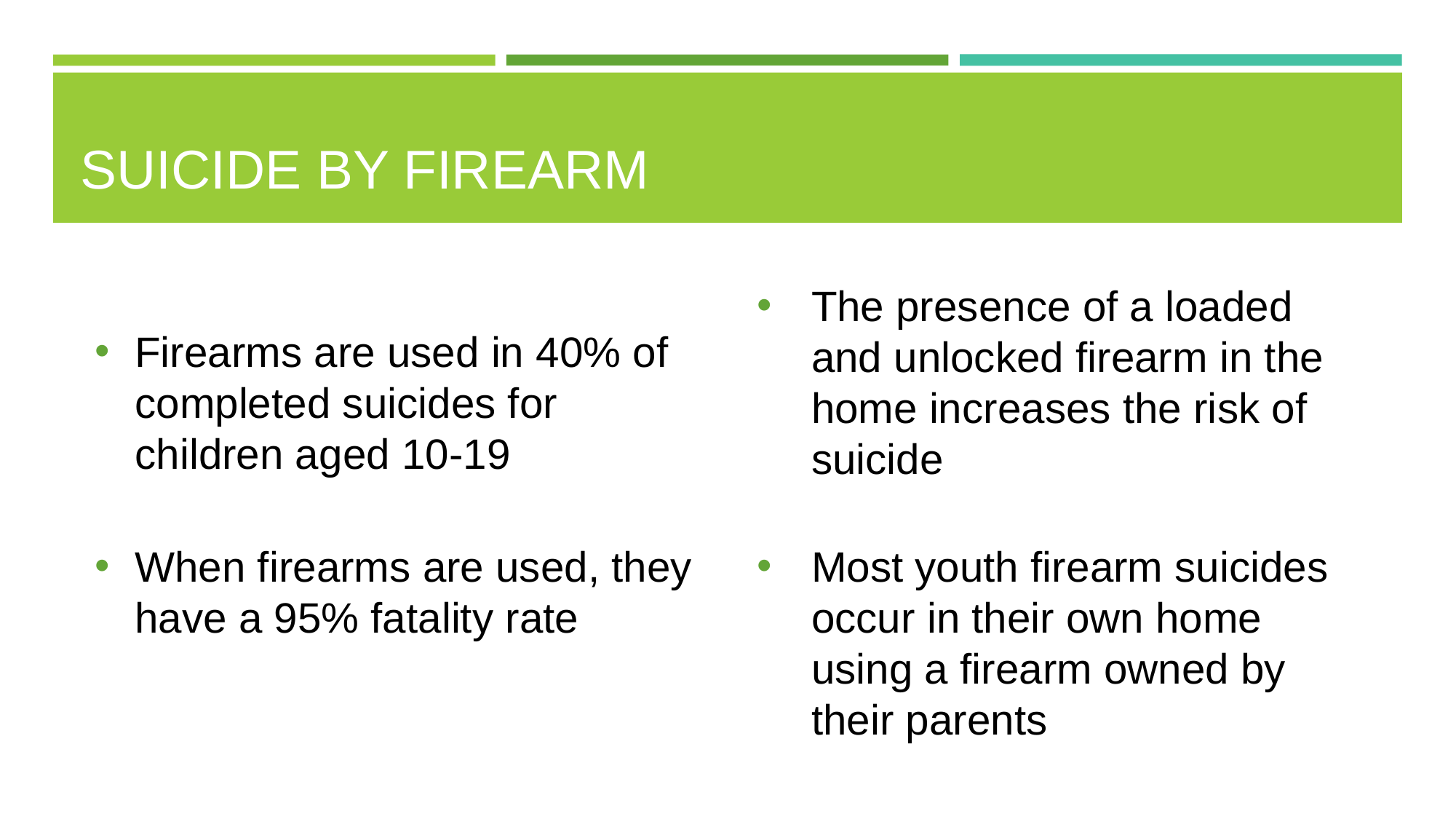

# SUICIDE BY FIREARM
Firearms are used in 40% of completed suicides for children aged 10-19
When firearms are used, they have a 95% fatality rate
The presence of a loaded and unlocked firearm in the home increases the risk of suicide
Most youth firearm suicides occur in their own home using a firearm owned by their parents

## Slide 16
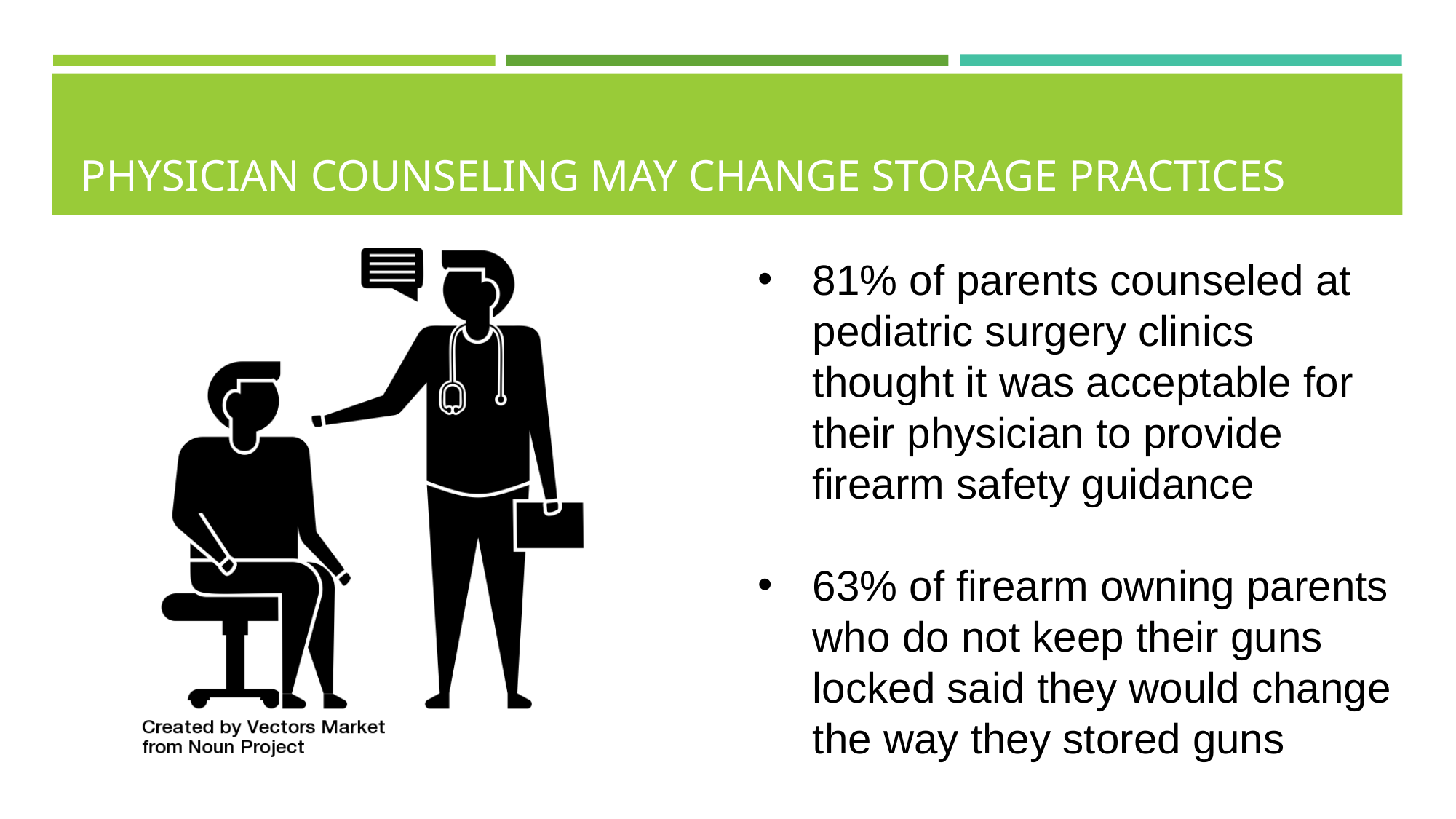

# PHYSICIAN COUNSELING MAY CHANGE STORAGE PRACTICES
81% of parents counseled at pediatric surgery clinics thought it was acceptable for their physician to provide firearm safety guidance
63% of firearm owning parents who do not keep their guns locked said they would change the way they stored guns

## Slide 17
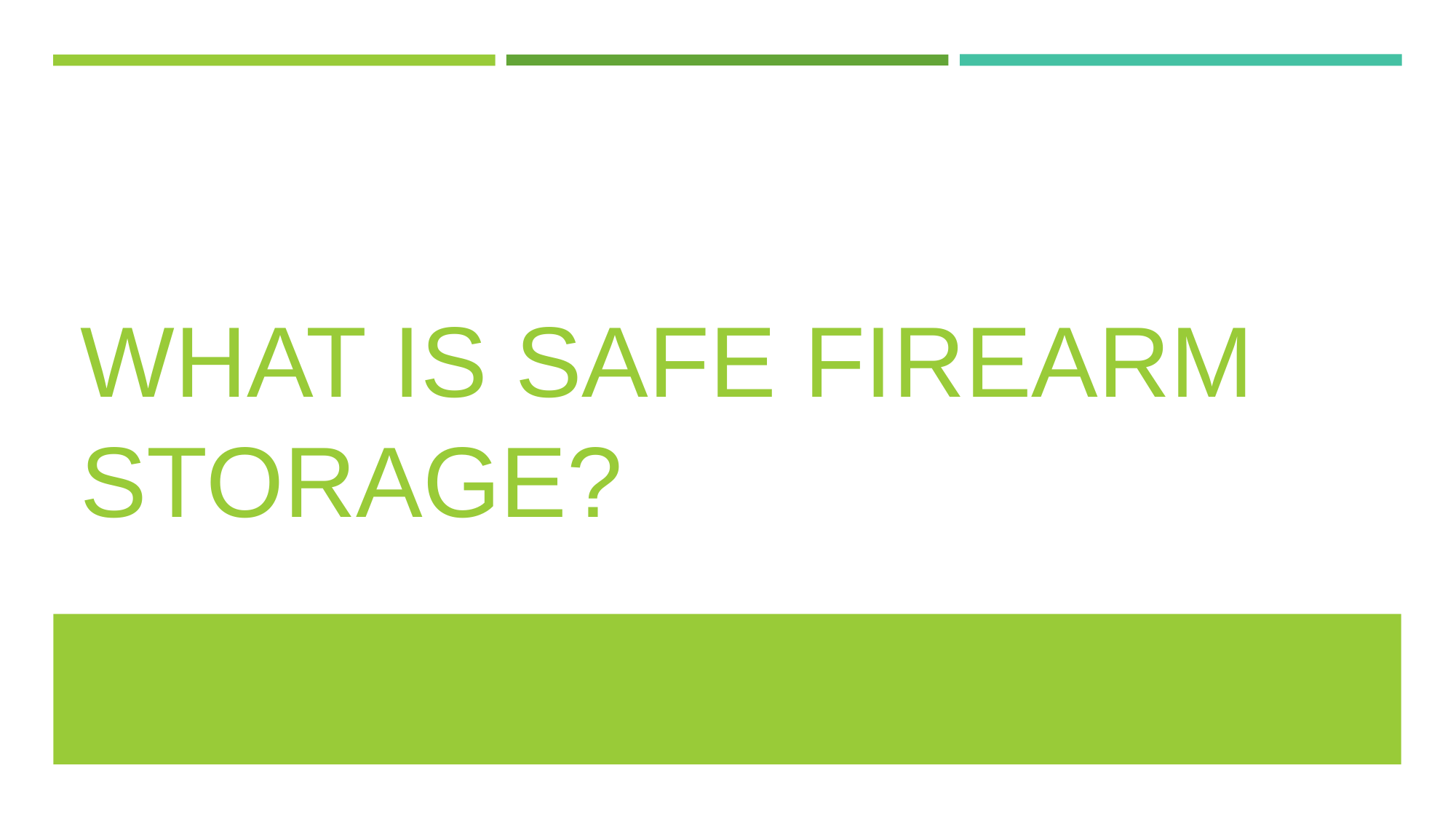

# WHAT IS SAFE FIREARM STORAGE?

## Slide 18
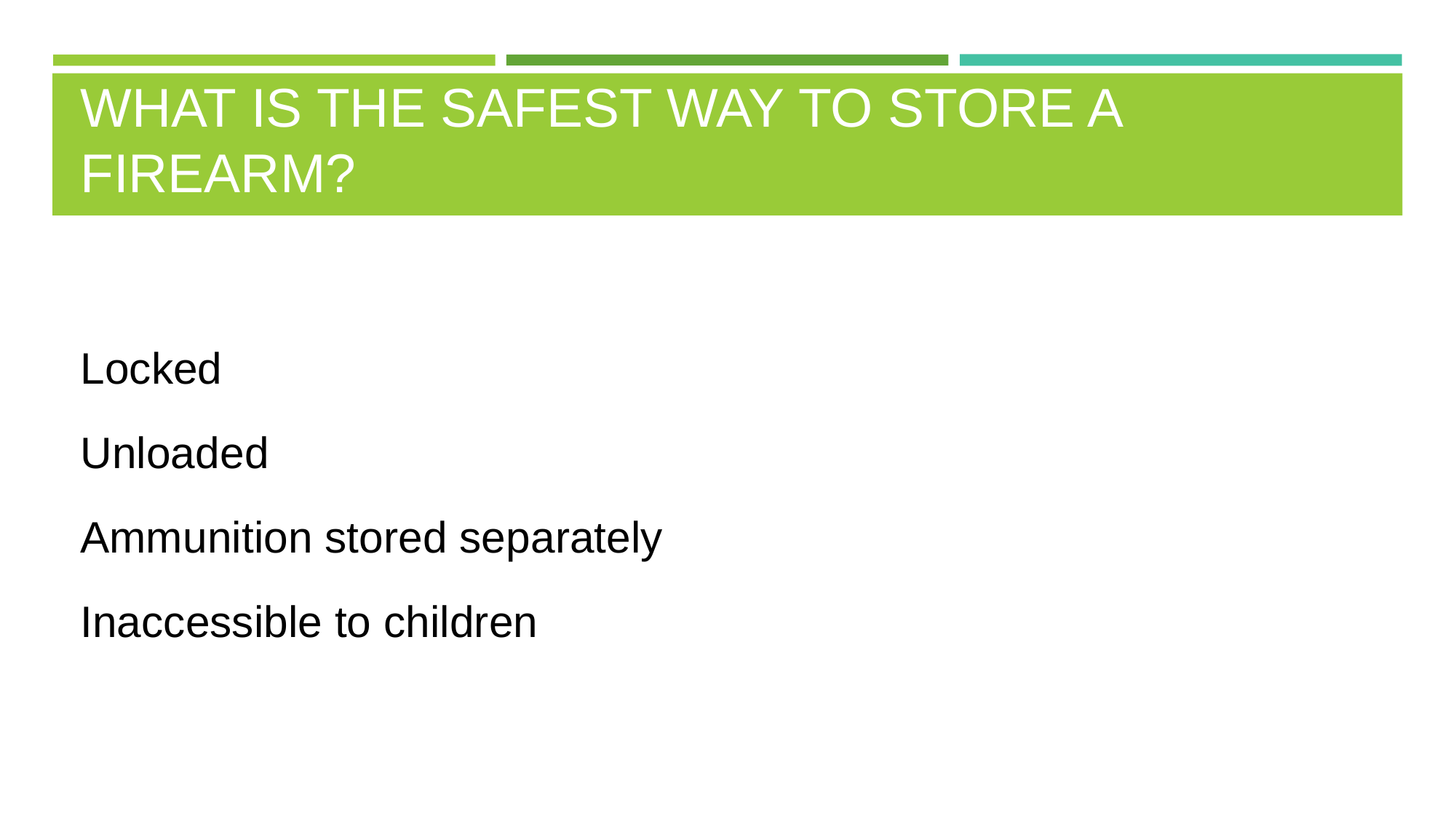

# WHAT IS THE SAFEST WAY TO STORE A FIREARM?
Locked
Unloaded
Ammunition stored separately
Inaccessible to children

## Slide 19
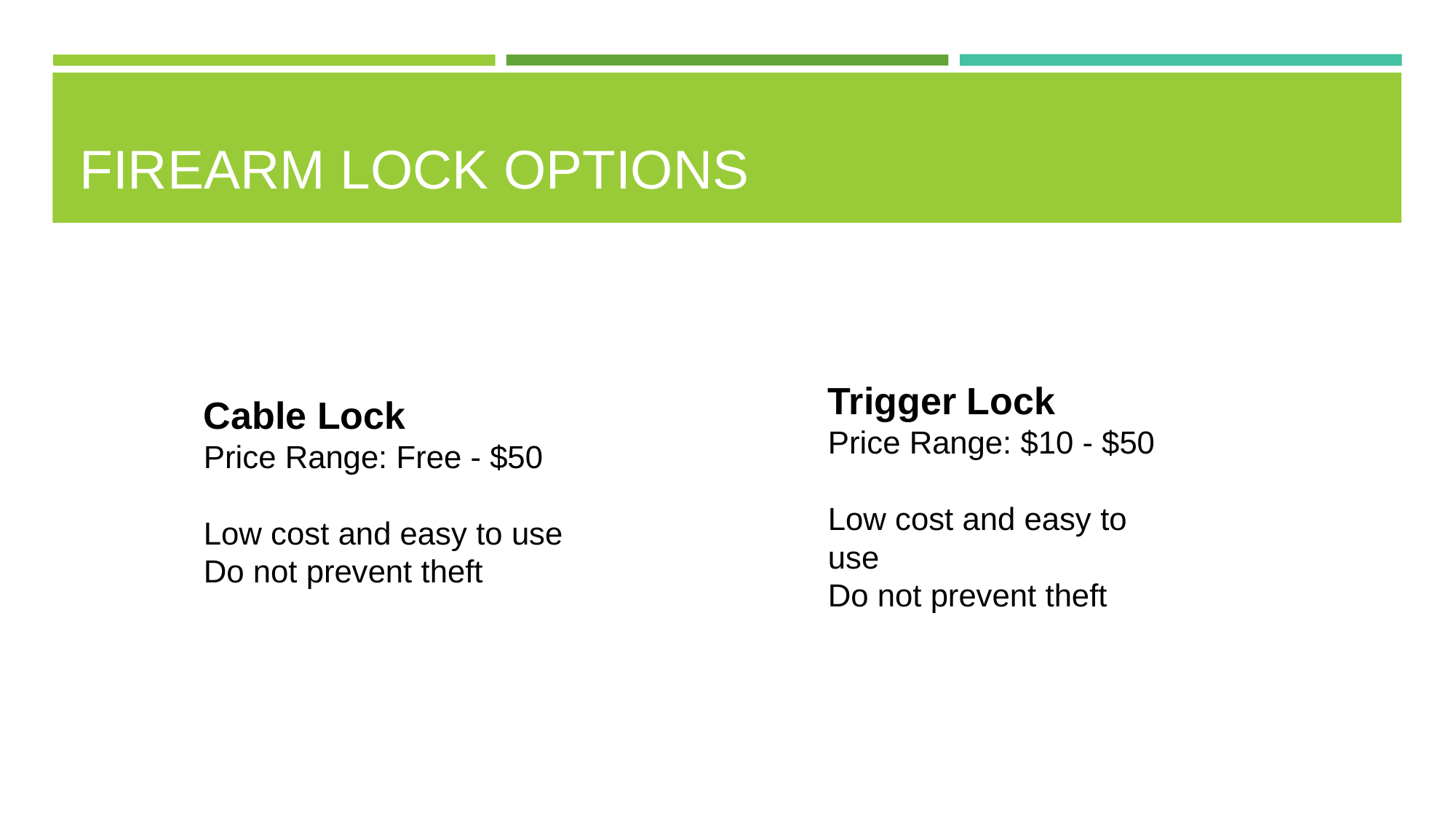

# FIREARM LOCK OPTIONS
Trigger Lock
Price Range: $10 - $50
Low cost and easy to use
Do not prevent theft
Cable Lock
Price Range: Free - $50
Low cost and easy to use
Do not prevent theft

## Slide 20
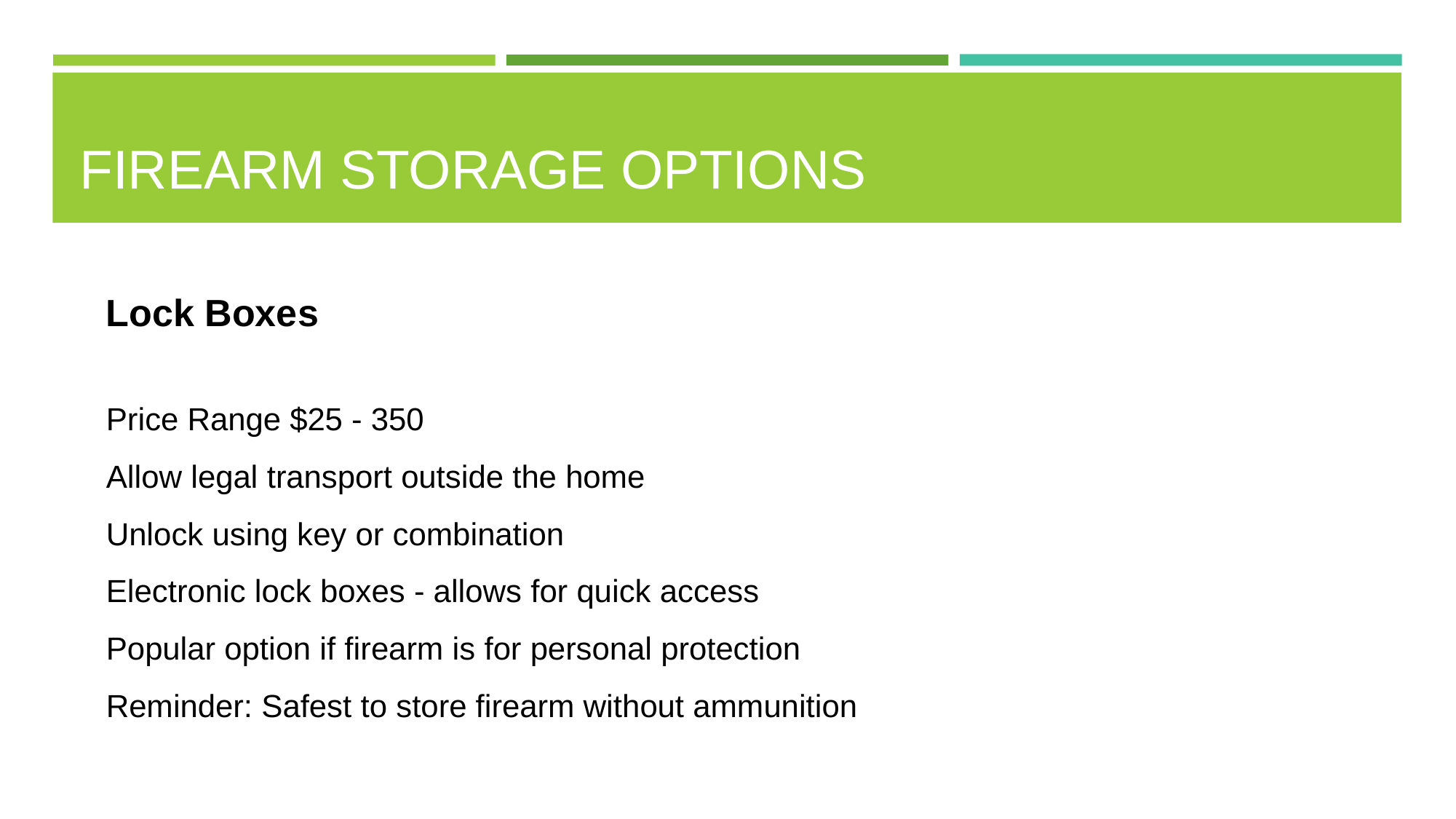

# FIREARM STORAGE OPTIONS
Lock Boxes
Price Range $25 - 350
Allow legal transport outside the home
Unlock using key or combination
Electronic lock boxes - allows for quick access
Popular option if firearm is for personal protection
Reminder: Safest to store firearm without ammunition

## Slide 21
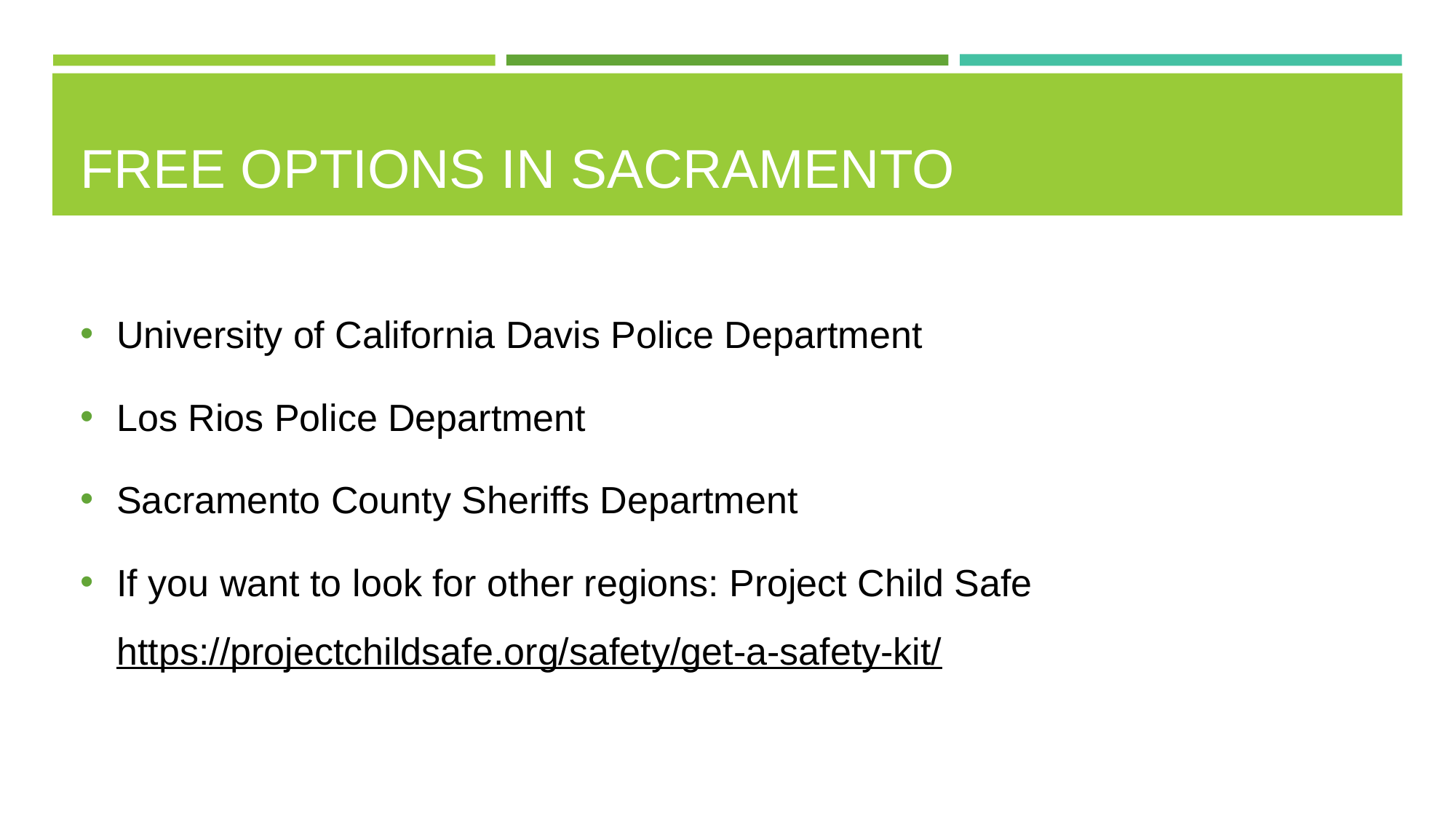

# FREE OPTIONS IN SACRAMENTO
University of California Davis Police Department
Los Rios Police Department
Sacramento County Sheriffs Department
If you want to look for other regions: Project Child Safe https://projectchildsafe.org/safety/get-a-safety-kit/

## Slide 22
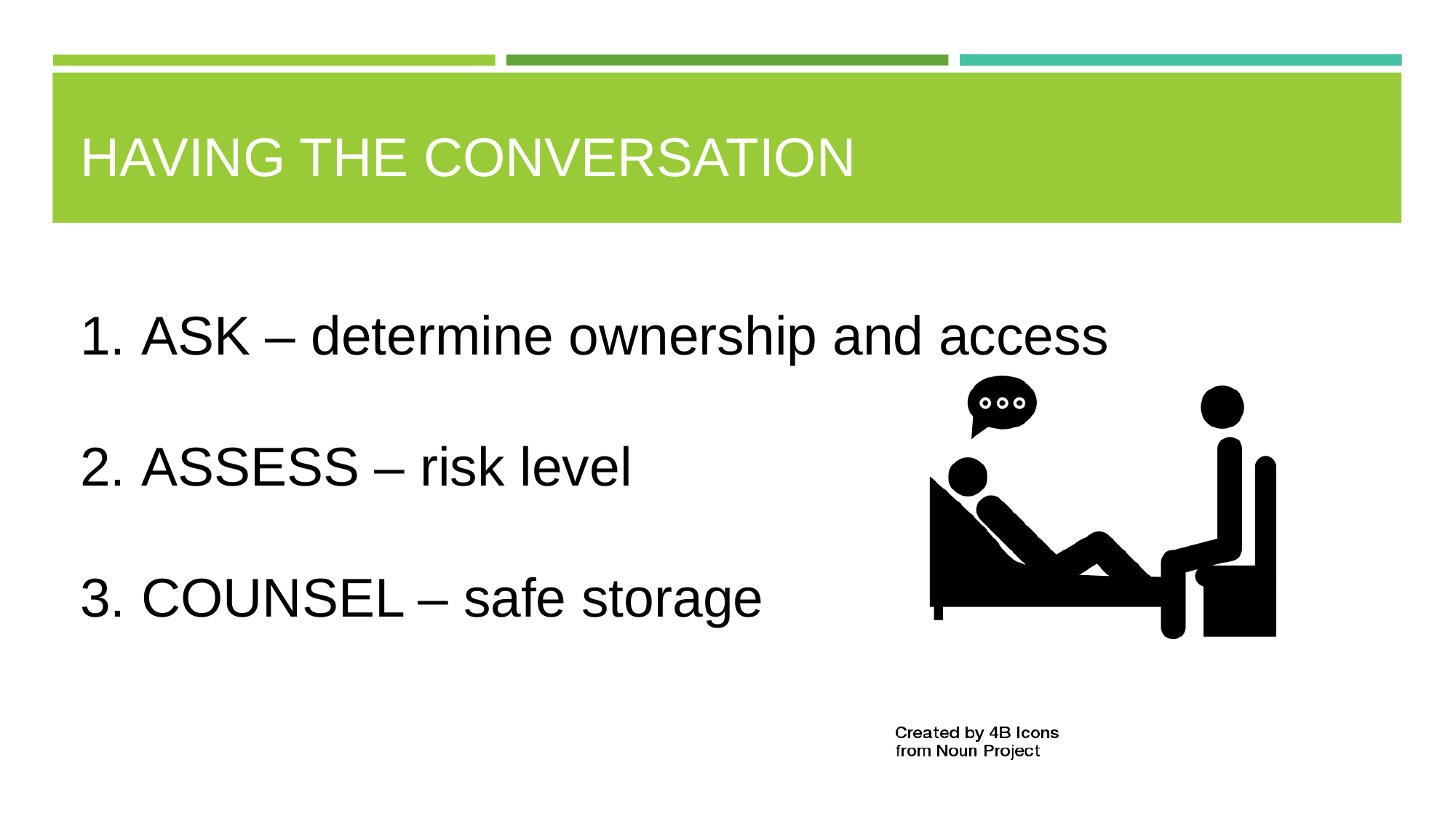

# HAVING THE CONVERSATION
ASK – determine ownership and access
ASSESS – risk level
COUNSEL – safe storage

## Slide 23
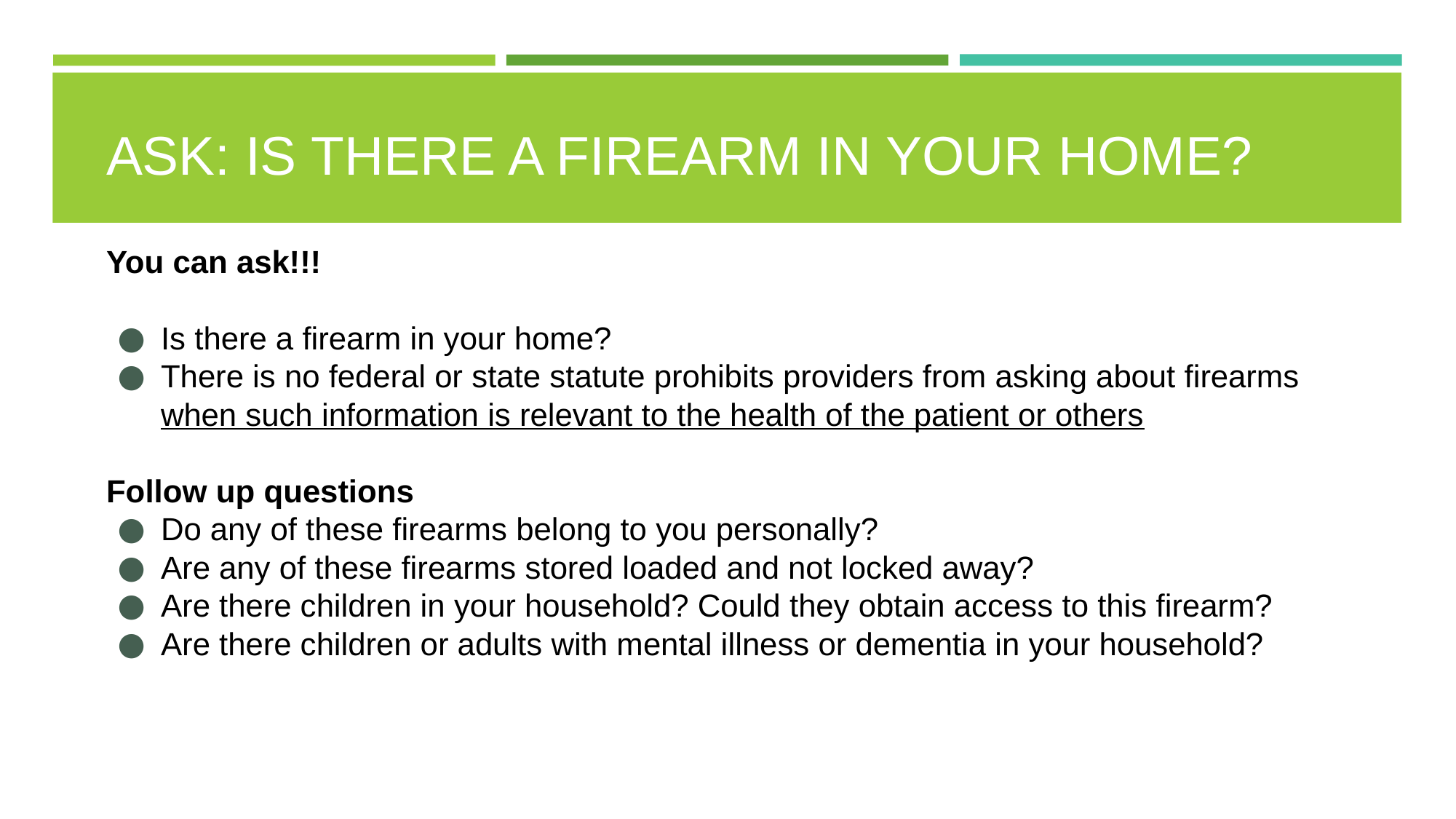

# ASK: IS THERE A FIREARM IN YOUR HOME?
You can ask!!!
Is there a firearm in your home?
There is no federal or state statute prohibits providers from asking about firearms when such information is relevant to the health of the patient or others
Follow up questions
Do any of these firearms belong to you personally?
Are any of these firearms stored loaded and not locked away?
Are there children in your household? Could they obtain access to this firearm?
Are there children or adults with mental illness or dementia in your household?

## Slide 24
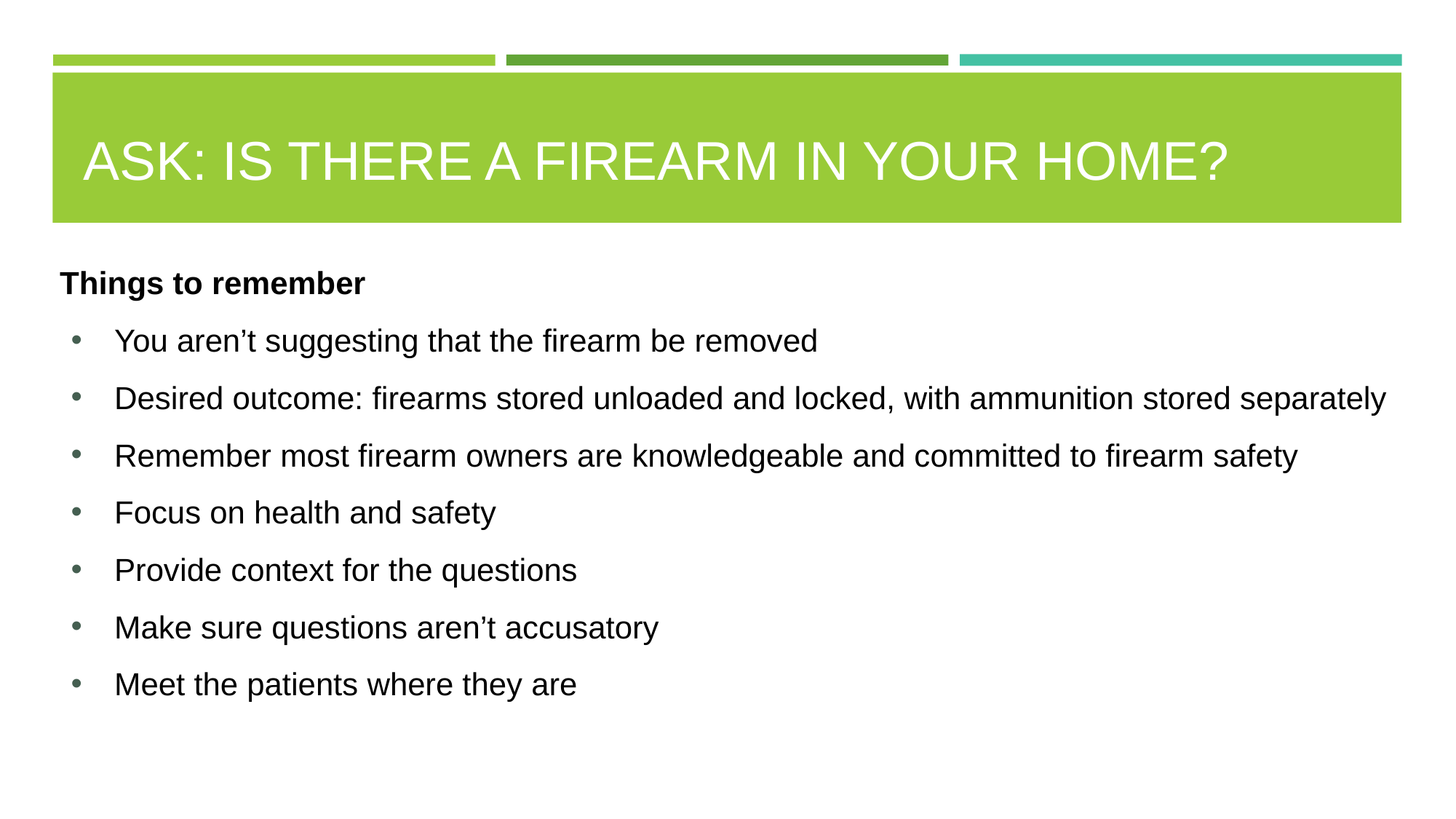

# ASK: IS THERE A FIREARM IN YOUR HOME?
Things to remember
You aren’t suggesting that the firearm be removed
Desired outcome: firearms stored unloaded and locked, with ammunition stored separately
Remember most firearm owners are knowledgeable and committed to firearm safety
Focus on health and safety
Provide context for the questions
Make sure questions aren’t accusatory
Meet the patients where they are

## Slide 25
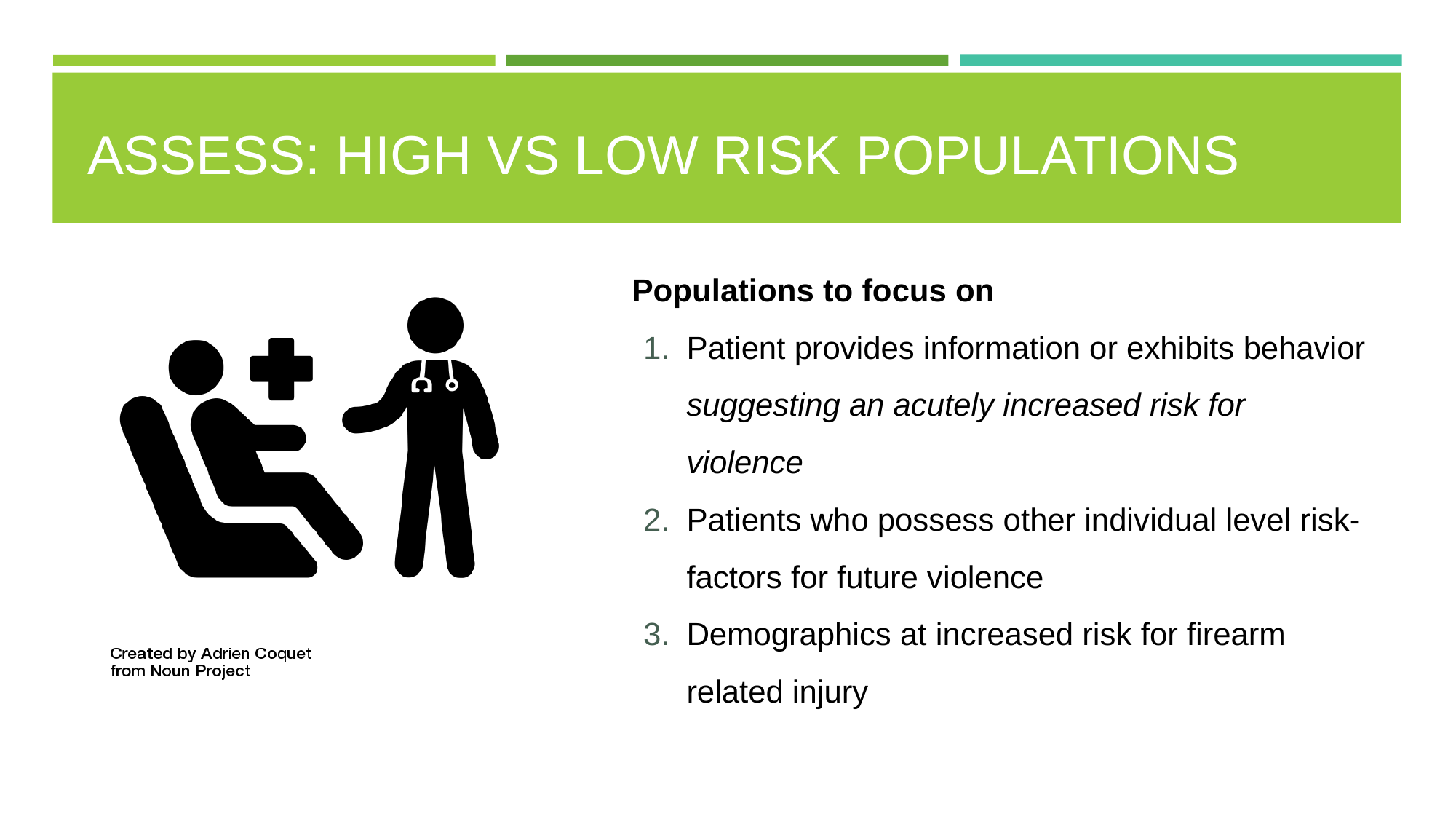

# ASSESS: HIGH VS LOW RISK POPULATIONS
Populations to focus on
Patient provides information or exhibits behavior suggesting an acutely increased risk for violence
Patients who possess other individual level risk-factors for future violence
Demographics at increased risk for firearm related injury

## Slide 26
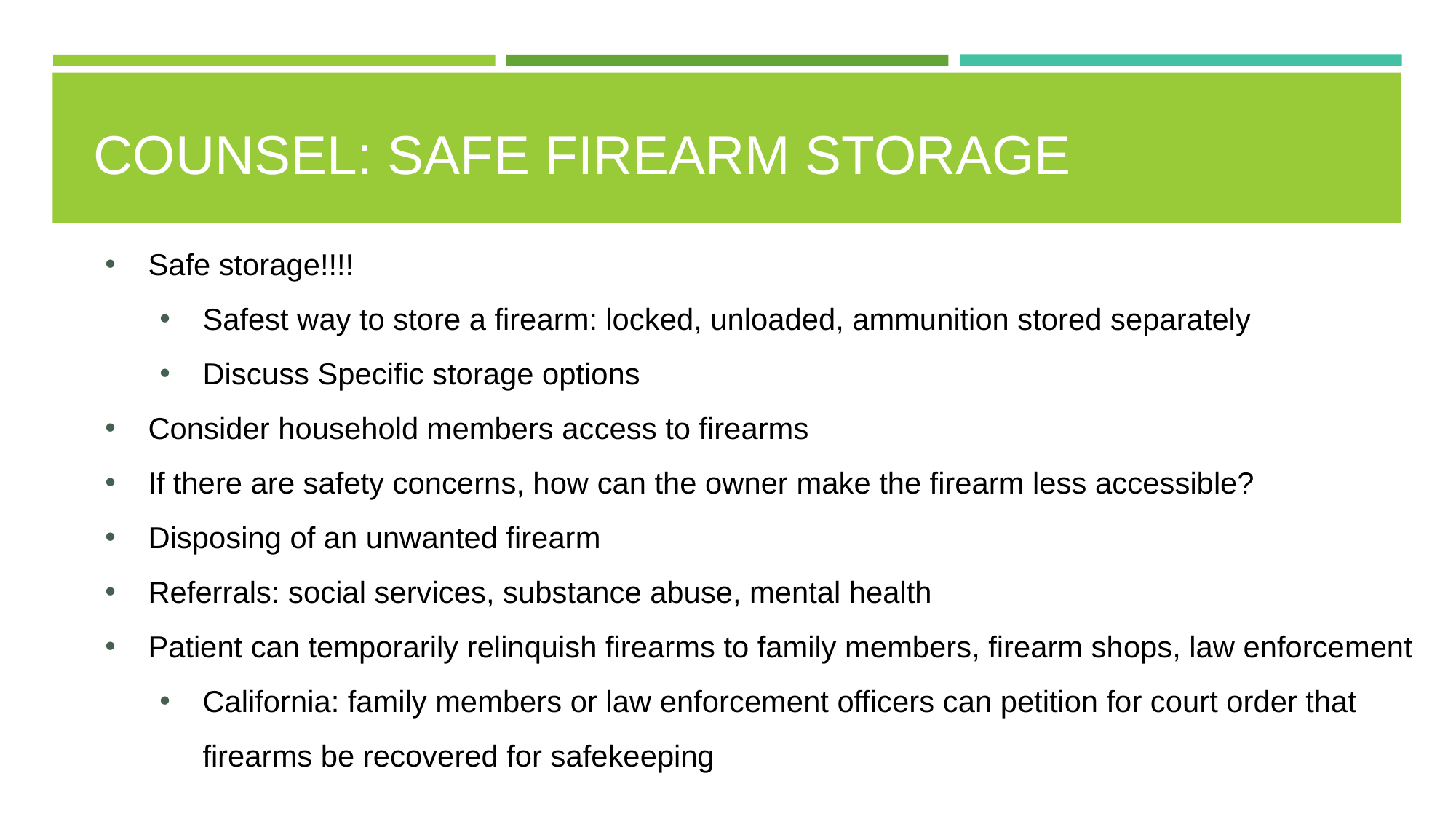

# COUNSEL: SAFE FIREARM STORAGE
Safe storage!!!!
Safest way to store a firearm: locked, unloaded, ammunition stored separately
Discuss Specific storage options
Consider household members access to firearms
If there are safety concerns, how can the owner make the firearm less accessible?
Disposing of an unwanted firearm
Referrals: social services, substance abuse, mental health
Patient can temporarily relinquish firearms to family members, firearm shops, law enforcement
California: family members or law enforcement officers can petition for court order that firearms be recovered for safekeeping

## Slide 27
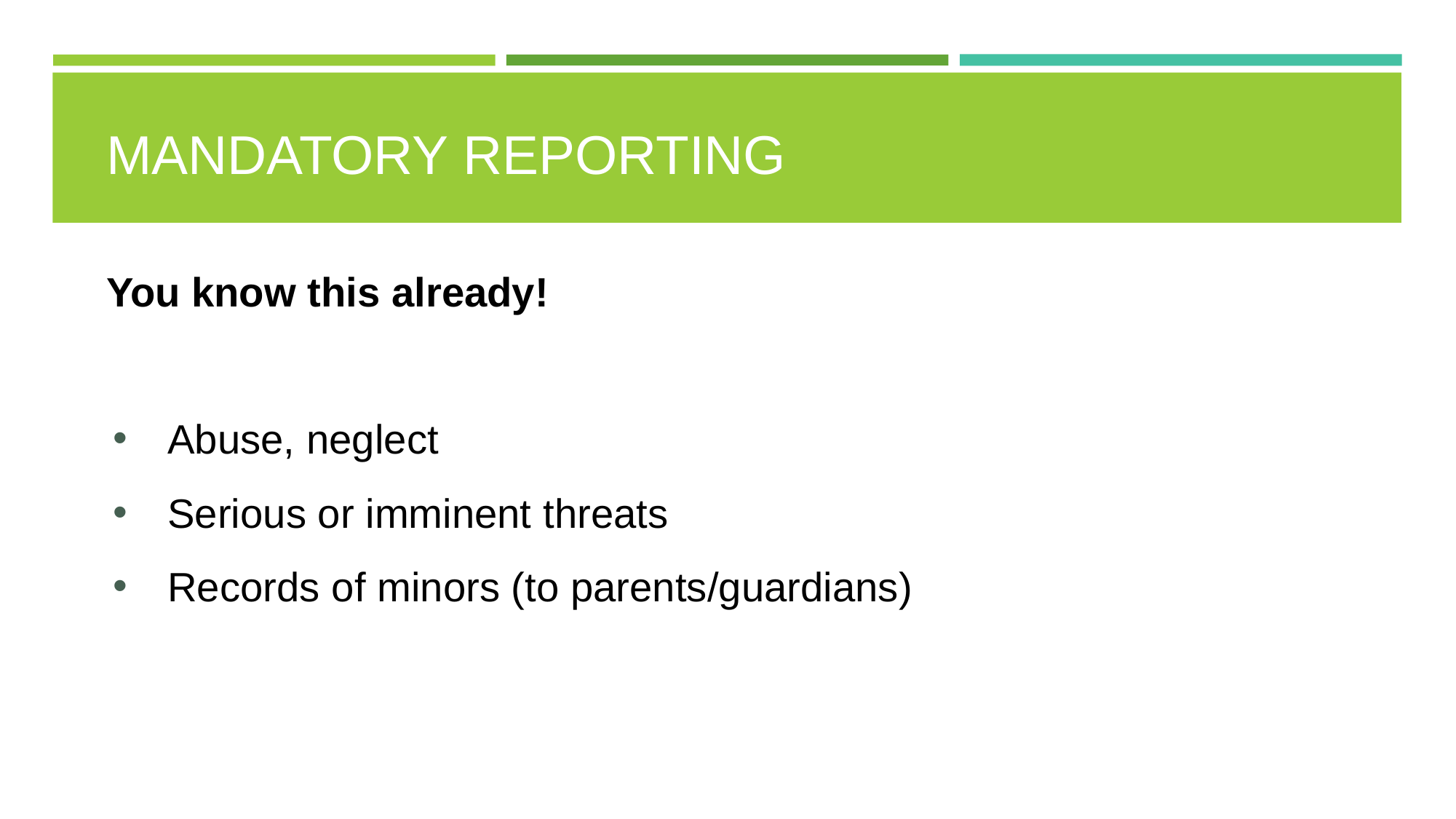

# MANDATORY REPORTING
You know this already!
Abuse, neglect
Serious or imminent threats
Records of minors (to parents/guardians)

## Slide 28
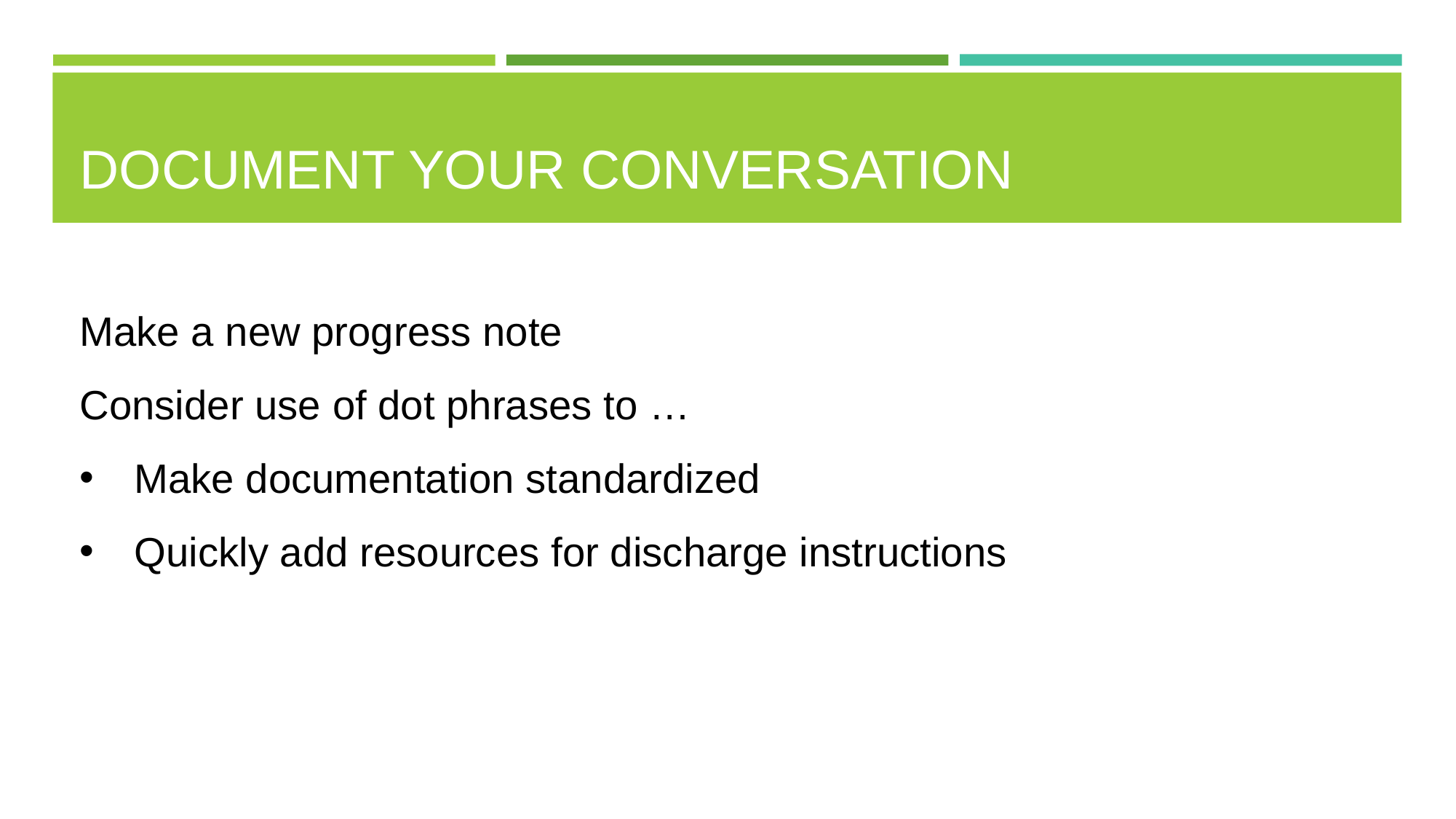

# DOCUMENT YOUR CONVERSATION
Make a new progress note
Consider use of dot phrases to …
Make documentation standardized
Quickly add resources for discharge instructions

## Slide 29
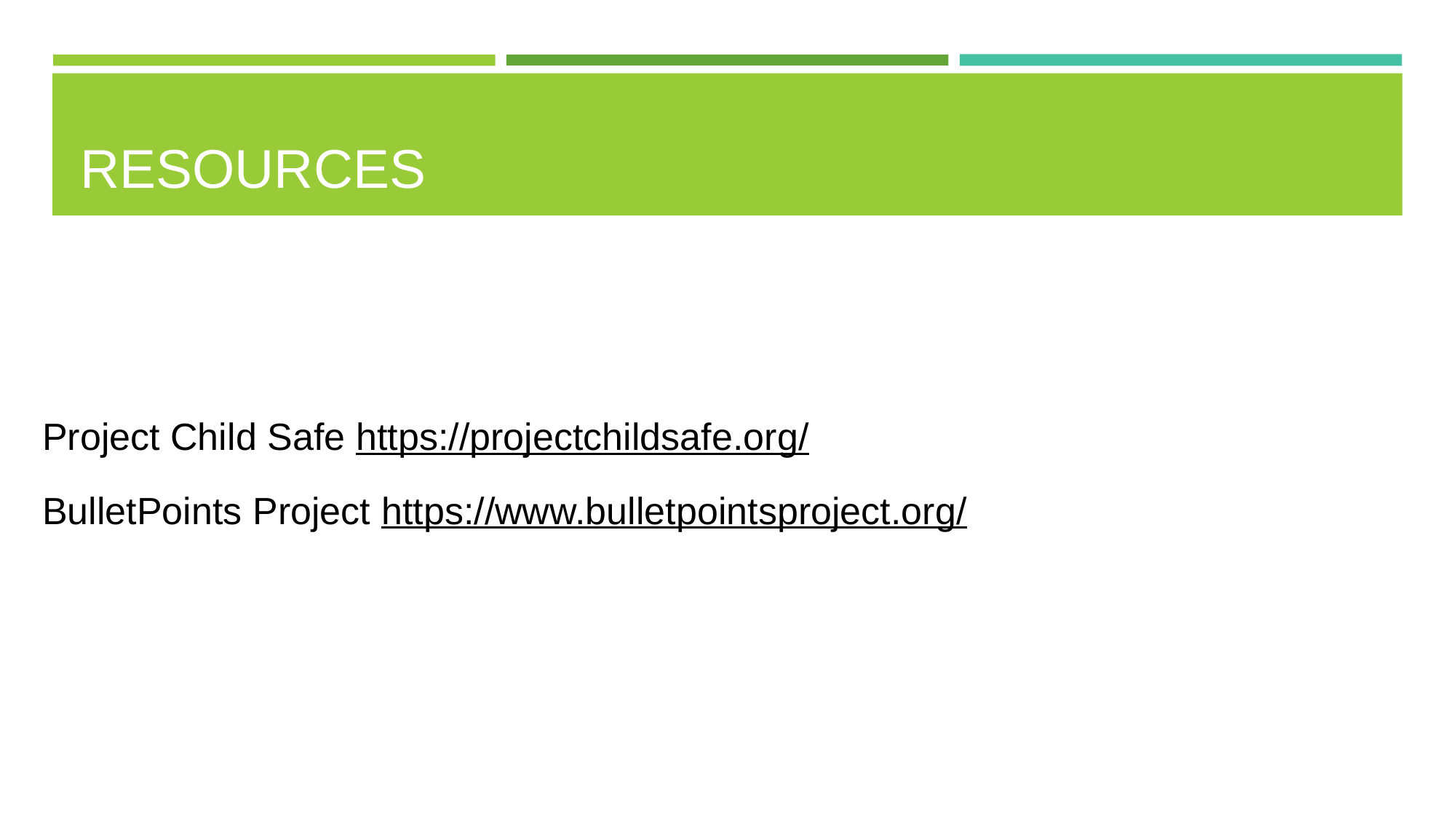

# RESOURCES
Project Child Safe https://projectchildsafe.org/
BulletPoints Project https://www.bulletpointsproject.org/
