# Supplementary material for: Firearm Safety Counseling for Patients: An Interactive Curriculum for Trauma Providers
Source: MedEdPORTAL. 2022 May 10;18:11237. doi: 10.15766/mep_2374-8265.11237 (PMC9085984; doi:10.15766/mep_2374-8265.11237)
Supplement: Supplementary file 1 — Safe Firearm Storage.pptxStandardized Patient Cases.docxPresentation of Standardized Patient Cases.docxPre- and Postsurveys.docx [file mep_2374-8265.11237-s001.zip › B. Standardized Patient Cases.docx]

Appendix B: *Standardized patients*

*Standardized patient - Richard*

Date: September 5, 2020

Primary Case Author: Sarah Stokes

Secondary Case Author: Nikia McFadden

Standardized Patient Educator: Sarah Stokes and Nikia McFadden

Name of Case: Suicide risk and firearms

Name of educational and or assessment activity: Safe firearm storage counseling

Patient Name: Richard

Chief Complaint: suicide attempt

Most likely Diagnosis and Differential with rationale from history and/or physical exam:

Firearm stored unlocked by a patient with a history of suicidal ideation

Challenge question:

Domains: Check all that apply

- Professionalism

X Communication and Interpersonal skills

- Medical History
- Physical exam
- Shared Decision Making

X Patient Education

- Clinical Reasoning
- Documentation
- Handoff
- Presentation
- Other:

Type and level of learner: Surgery or Emergency Medicine Resident or Trauma Nurse Practitioner

Case Objectives: please list specific objectives for each of the domains you have checked above:

1. Learner will evaluate patient risks for firearm injury

2. Learner will counsel patient on safe firearm storage

| SETTING: outpatient, in patient, ED, home, nursing home, rehab, group etc. | Inpatient |
| --- | --- |
| PATIENT PROFILE: Information about the “patient” that helps select an SP and helps the learner get an understanding of them as a person. SP will know more information about the patient than learner will ever ask but allows SP to portray a fully developed patient personality. If none of the items below are particulars for the case please write “all may be used.” | |
| Age range | 60-70 years |
| Religious/spiritual background | All may be used |
| Sex (e.g., male, female, intersex, transwoman, transman) | Male |
| Sexual Orientation (e.g., heterosexual, lesbian, gay, bisexual, pansexual, queer, asexual) | Heterosexual |
| Gender expression (e.g., man, woman, gender queer) | Man |
| Race/ethnicity: | White, non-Hispanic |
| Physical description (e.g., BMI, height range) | All may be used |
| Physical limitations | All may be used |
| Patient appearance (e.g., disheveled, hospital gown, business casual, casual) | Hospital gown |
| Moulage + location (e.g., none, bruises, scars, body piercing, tattoos) | Healing incisions on bilateral wrists |
| Affect (e.g., pleasant, cooperative) | Cooperative, anxious about leaving the hospital soon |
| Family group (e.g., who is family, who they live with) | Lives alone, adult daughter previously lived nearby but moved out of state last year |
| Education | All may be used |
| Level of health literacy | Low |
| Employment, if any - present and past, noting any current stresses | Retired |
| Home/homeless - type of dwelling, number of stories, owned or rented | Lives in owned or rented home alone |
| Financial situation- any current stresses | No acute stresses, able to afford housing, food, clothing, some additional money |
| Insurance Status (e.g., un/under/insured, public/private, HMO/PPO) | Medicare |
| Habits (i.e., diet, exercise, caffeine, smoking, alcohol, drugs) | Drinks 2-3 beers a night, no drug use |
| Activities (i.e., hobbies, sports, clubs, friends) | Some friends but less socially active since retirement, likes to go for short hikes with friends |
| Typical day - what is the usual daily routine | Home projects, woodwork, sees friends every 1-2 weeks |

| CASE INFORMATION | |
| --- | --- |
| Chief Concern: What the patient will say when greeted by the student. The patient’s primary reason for seeking medical care often stated in his/own words. | Thank you so much for taking care of me. My daughter is flying in to pick me up today. What time do you think I will be ready to leave? |
| Additional Concerns: Other, if any, concerns the patient has today (i.e., symptoms, requests, expectations, etc.) that will become part of set agenda. | If asked ‘Why is firearm stored this way?’ Everyone in my family stores guns like this.  If counseled on handing your gun over to a relative: Ask ‘does this mean I will have a record on me that I am crazy?’  If counseled on storing your gun at a gun range: ‘I don’t think I can afford that’  If asked if you might attempt to hurt yourself again: ‘I don’t think so. I feel good right now and am planning on working with a psychiatrist’ |
|  | |
| THE PATIENT STORY: The SP will be asked to tell their symptom story and the personal and emotion impact for each of their concerns. You will want to write this is the patient voice. The symptom story should be able to answer this question: “Tell me more about [chief concern/additional concern], starting at the beginning and bringing me up to now.”  The personal context should be able to answer questions concerning the broader personal/psychosocial context of symptoms, especially the patient beliefs/attributions.  The emotional context should be able to ask how are you doing with this, how does this make you feel, how has this affected you emotionally? IMPACT: How has this affected your life? How has this been for your family? | I’m embarrassed to tell the story. I found out that my ex-wife was getting remarried. I wasn’t really thinking and cut my wrists. As soon as I had done it I called 911. I don’t want to die. The doctors here helped me and fixed up my wounds. I’ve talked to all the shrinks here and they say I’m good to go. The doctor this morning said I could go home today, when can I leave? |
| HISTORY OF PRESENT ILLNESS: Although some of the HPI will be given in the patient’s symptom story, the learners will expand the story during the direct question section. Below describe the detailed history, usually about the chief concern, which the student must develop in order to make a useful assessment of the problem: | |
| 65-year-old man taken to the hospital by ambulance after attempted to cut your wrists. Live alone, divorced, and estranged from your ex-wife. He received news that your ex-wife was planning on remarrying on the day of your injury. After attempting to cut your wrists he called 911. On arrival he was found to have superficial injuries, wounds were washed out and the lacerations repaired. He has been evaluated by psychiatry and determined to not be at acute risk of suicide and in coordination with social work determined to be safe to discharge home.  He owns a single handgun that you keep in the bedside table. It is stored in the same place as the ammunition, and not locked. His father always had a gun, so he has one too. He do not have many visitors, though occasionally his daughter and grandchild come to visit. His do not think your grandchild knows about the gun or would have access to it. | |
| Onset (when; gradual or sudden) | History of depression, superficial wrist laceration self inflicted |
| Setting (what was going on or where was patient when symptoms first noticed?) | Patient self-inflicted superficial wrist laceration |
| Duration (how long) | N/A |
| Time relationships (frequency, constant or intermittent) | N/A |
| Location | N/A |
| Radiation | N/A |
| Quality | N/A |
| Amount | N/A |
| Aggravated by what | N/A |
| Relieved by what | N/A |
| Associated with what | N/A |
| Attitude (what does the patient think is the problem, and how does he/she feel about it) | Acutely stressed by learning news of ex-wife getting remarried. Now feels fine, reports no suicidal ideation. |
| Overall course |  |
| REVIEW OF SYSTEMS: Significant positives and negatives | |
| Has firearm (handgun) at home | Does not report active suicidal ideation. |
| Firearm is stored with ammunition in bedside drawer. |  |
| Does not use firearm lock and drawer is usually unlocked. |  |
| Daughter and grandson occasionally visit. Patient does not think grandson knows where firearm is. |  |
|  | |
| Past medical history |  |
| Medication allergies (Name and reaction) | None |
| Environmental allergies (Name and reaction) | None |
| Illnesses | Depression, hypertension |
| Vaccinations | Up to date |
| Surgeries | None |
| Accidents/ injuries/ trauma | None prior to current |
| Hospitalization | None prior to current |
|  | |
| Inclusive sexual and reproductive history | |
| Sexual practices  Sexual partners  Protection: Use of safer sex practices  Use of birth control if appropriate  Risk of intimate partner violence | Heterosexual  None current  None with ex-wife  N/A  No |
| Ob/GYN HISTORY | N/A |
| Medications | Prescription/dose/reason - None  Over the counter/dose/reason - None  Herbs/supplements/dose/reason - None  Other: |
| Immunizations | X Tetanus  X Flu  X Hepatitis  X Pneumovax  X HPV   - Other |
| Tobacco products:   - Cigarettes - Cigar - Pipe - Chew - E-cigarettes | X Never   - Past- year started/year quit - Current   - Quantity   - # of years |
| Alcohol  X Beer   - Wine - Liquor - Other | - Never - Past- year started/year quit   X Current   - - Quantity – 2-3 per night   - # of years – increased in past 3 years |
| Drugs   - Weed - Cocaine - Heroin - Meth - Other - IV - Inhalants - Other | X Never   - Past- year started/year quit - Current   - Quantity - # of years |
| Diet (describe) | Any |
| Exercise (describe) | Occasional hikes |
| List any other important social history or information important to this case | Divorced and estranged from ex-wife. Daughter moved out of state about a year ago. She is on her way back to pick him up from the hospital and plans to stay for a few weeks. She is bringing her 6 year old son. |
| Family history |  |
| Mother, Father, Siblings, Grandparents, and other significant findings. | Any |
|  |  |
| Physical Exam- List exam maneuvers expected for this case and any abnormal findings that SP will simulate. (tenderness, hyper-hypo reflex, rebound, weakness etc. )  No physical exam expected. | |
| PHYSICAL EXAM FINDINGS |  |
| 1. Written in layman’s terms |  |
| 1. General appearance- affect, appearance, position of patient at opening (i.e. sitting, laying down, holding abdomen etc.) |  |
| 1. Vital signs |  |
| 1. Specific findings and affect |  |
| 1. Response to certain physical movements |  |
|  |  |
| DIAGNOSIS AND DIFFERENTIAL |  |
| Diagnosis with support from positive and negative history and PE findings | Patient with recent suicide attempt and history of depression who owns a firearm and stores it unlocked. Additionally, the patient’s grandson, who is 6 years old, will be staying with the patient for a few weeks. Risk for patient for future self-inflicted firearm injury, and risk for grandson for unintentional injury. |
| Differential with support from positive and negative history and PE findings |  |
|  |  |
| MANAGEMENT OR DIAGNOSTIC PLAN | Counsel patient on safe firearm storage, describing why the patient may be at higher risk. Describe options for safe firearm storage. |
|  |  |
| PROFESSIONALISM ISSUES OR CHALLENGES: |  |

*Standardized patient – Anna and Max*

Date: September 5, 2020

Primary Case Author: Sarah Stokes

Secondary Case Author: Nikia McFadden

Standardized Patient Educator: Sarah Stokes and Nikia McFadden

Name of Case: Unintentional injury risk and firearms

Name of educational and or assessment activity: Safe firearm storage counseling

Patient Name: Max, history obtained from mother, Anna

Chief Complaint: self-inflected unintentional firearm injury

Most likely Diagnosis and Differential with rationale from history and/or physical exam:

Firearm stored unlocked by a patient with a history of suicidal ideation

Challenge question:

Domains: Check all that apply

- Professionalism

X Communication and Interpersonal skills

- Medical History
- Physical exam
- Shared Decision Making

X Patient Education

- Clinical Reasoning
- Documentation
- Handoff
- Presentation
- Other:

Type and level of learner: Surgery or Emergency Medicine Resident or Trauma Nurse Practitioner

Case Objectives: please list specific objectives for each of the domains you have checked above:

1. Learner will evaluate patient risks for firearm injury

2. Learner will counsel patient on safe firearm storage

| SETTING: outpatient, in patient, ED, home, nursing home, rehab, group etc. | Inpatient |
| --- | --- |
| PATIENT PROFILE: Information about the “patient” that helps select an SP and helps the learner get an understanding of them as a person. SP will know more information about the patient than learner will ever ask but allows SP to portray a fully developed patient personality. If none of the items below are particulars for the case please write “all may be used.” | |
| Age range | 20s-30s (mother) (son is 6 years old, will not be part of conversation) |
| Religious/spiritual background | Any |
| Sex (e.g., male, female, intersex, transwoman, transman) | Female |
| Sexual Orientation (e.g., heterosexual, lesbian, gay, bisexual, pansexual, queer, asexual) | Heterosexual |
| Gender expression (e.g., man, woman, gender queer) | Woman |
| Race/ethnicity: | Any |
| Physical description (e.g., BMI, height range) | Any |
| Physical limitations | None |
| Patient appearance (e.g., disheveled, hospital gown, business casual, casual) | Casual |
| Moulage + location (e.g., none, bruises, scars, body piercing, tattoos) | None |
| Affect (e.g., pleasant, cooperative) | Cooperative |
| Family group (e.g., who is family, who they live with) | Family – mother, father, 3 children (6 years, 8 years and 10 years), all live together |
| Education | High school |
| Level of health literacy | High |
| Employment, if any - present and past, noting any current stresses | High school teacher |
| Home/homeless - type of dwelling, number of stories, owned or rented | Farm, owned |
| Financial situation- any current stresses | No acute stresses, comfortable |
| Insurance Status (e.g., un/under/insured, public/private, HMO/PPO) | Insured, private |
| Habits (i.e., diet, exercise, caffeine, smoking, alcohol, drugs) | Occasional alcohol use for Anna and her husband (1-2 beers or glasses of wine once a week), none for children |
| Activities (i.e., hobbies, sports, clubs, friends) | Any |
| Typical day - what is the usual daily routine | Anna at work from 7-4pm, husband often out of town for work, children at school during day |

| CASE INFORMATION | |
| --- | --- |
| Chief Concern: What the patient will say when greeted by the student. The patient’s primary reason for seeking medical care often stated in his/own words. | I am so happy my son is doing better. When will we be able to go home? |
| Additional Concerns: Other, if any, concerns the patient has today (i.e., symptoms, requests, expectations, etc.) that will become part of set agenda. | When the resident/NP brings up safe gun storage you are open to the conversation, but think you probably know a lot more about guns than they do and are a little irritated as you feel that they are judging you without having a true understanding of your life. You live on your family farm, and everyone in your family hunts. You have grown up with guns, your kids are growing up with guns, and you are careful to teach them about safety (you have started talking to your 6-year-old about how to handle a gun - you have told him to never point it at himself or others, always assume it is loaded).  If told to store guns locked and unloaded, with ammunition stored in a separate place - share concerns about household safety and that you store the gun in the kitchen for easy access if you need to defend your home. Your husband is often away for work and you are anxious about being home alone with only the kids.  If asked:  Why is the gun in the kitchen stored in this manner? ‘I want quick access if someone breaks in. I live in a very rural area and am concerned that the police would take a long time to respond if I called them.’ |
|  | |
| THE PATIENT STORY: The SP will be asked to tell their symptom story and the personal and emotion impact for each of their concerns. You will want to write this is the patient voice. The symptom story should be able to answer this question: “Tell me more about [chief concern/additional concern], starting at the beginning and bringing me up to now.”  The personal context should be able to answer questions concerning the broader personal/psychosocial context of symptoms, especially the patient beliefs/attributions.  The emotional context should be able to ask how are you doing with this, how does this make you feel, how has this affected you emotionally? IMPACT: How has this affected your life? How has this been for your family? | My son was playing in the living room with his sister. I went upstairs to finish some grading, I just had a lot to do and needed a little quiet. I can’t believe I left them alone. I was only up there for about 10 minutes, I heard a gunshot and I ran downstairs. I saw my son bleeding, with the gun on the ground. I called an ambulance and they came really fast. I still can’t forgive myself, but I’m happy the doctors here have taken good care of him. Are we able to go home today? |
| HISTORY OF PRESENT ILLNESS: Although some of the HPI will be given in the patient’s symptom story, the learners will expand the story during the direct question section. Below describe the detailed history, usually about the chief concern, which the student must develop in order to make a useful assessment of the problem:  6-year-old boy (Max) was playing alone at home. He found his father’s gun and shot himself in the arm. He has gone to the operating room for a washout of the injury and is now in recovery. He is doing well and is ready for discharge. | |
|  | |
| Onset (when; gradual or sudden) | Acute unintentional self inflicted injury |
| Setting (what was going on or where was patient when symptoms first noticed?) | Acute unintentional self inflicted injury |
| Duration (how long) | N/A |
| Time relationships (frequency, constant or intermittent) | Single injury |
| Location | Arm |
| Radiation | N/A |
| Quality | N/A |
| Amount | N/A |
| Aggravated by what | N/A |
| Relieved by what | N/A |
| Associated with what | N/A |
| Attitude (what does the patient think is the problem, and how does he/she feel about it) | Concerned about the health of child. Happy with the hospital and the care that child has received so far, but anxious to get home. |
| Overall course |  |
| REVIEW OF SYSTEMS: Significant positives and negatives | |
| 3 guns in house. Two are used for hunting and these are kept locked in the garage. A third gun that he stores in a cupboard above the refrigerator. The gun is stored loaded.  The cupboard is locked, and the keys are kept in a kitchen drawer. | Children occasionally have friends over to play, parents typically supervise them throughout their playtime, sometimes stepping away to make dinner, but they are always within earshot |
| Parents previously did not think children knew where firearm was. | Other visitors to the house are grandparents, no history of depression or dementia. |
| No history of depression in children or parents. |  |
|  |  |
|  | |
| Past medical history – for sone Max |  |
| Medication allergies (Name and reaction) | None |
| Environmental allergies (Name and reaction) | None |
| Illnesses | None |
| Vaccinations | Up to date |
| Surgeries | None prior to current |
| Accidents/ injuries/ trauma | None prior to current |
| Hospitalization | None prior to current |
|  | |
| Inclusive sexual and reproductive history – for 6 year old son, N/A | |
| Sexual practices  Sexual partners  Protection: Use of safer sex practices  Use of birth control if appropriate  Risk of intimate partner violence |  |
| Ob/GYN HISTORY | Age of onset of menses  Age of menopause  Number of pregnancies  Number of live births  Number of miscarriages  Number of abortions |
| Medications | Prescription/dose/reason - none  Over the counter/dose/reason - none  Herbs/supplements/dose/reason - none  Other: |
| Immunizations | X Tetanus   - Flu   X Hepatitis   - Pneumovax - HPV - Other |
| Tobacco products:   - Cigarettes - Cigar - Pipe - Chew - E-cigarettes | X Never (for son and parents)   - Past- year started/year quit - Current   - Quantity   - # of years |
| Alcohol   - Beer - Wine - Liquor - Other | X Never (for son)   - Past- year started/year quit   X Current (for parents)   - - Quantity – 1-2 beers or glasses of wine once a week   - # of years – 10 years |
| Drugs   - Weed - Cocaine - Heroin - Meth - Other - IV - Inhalants - Other | X Never (for son and parents)   - Past- year started/year quit - Current   - Quantity - # of years |
| Diet (describe) | Any |
| Exercise (describe) | Any |
| List any other important social history or information important to this case | Husband frequently away for work, family lives in rural area and mother concerned about home safety |
| Family history |  |
| Mother, Father, Siblings, Grandparents, and other significant findings. | None |
|  |  |
| Physical Exam- List exam maneuvers expected for this case and any abnormal findings that SP will simulate. (tenderness, hyper-hypo reflex, rebound, weakness etc. )  No physical exam. | |
| PHYSICAL EXAM FINDINGS |  |
| 1. Written in layman’s terms |  |
| 1. General appearance- affect, appearance, position of patient at opening (i.e. sitting, laying down, holding abdomen etc.) |  |
| 1. Vital signs |  |
| 1. Specific findings and affect |  |
| 1. Response to certain physical movements |  |
|  |  |
| DIAGNOSIS AND DIFFERENTIAL |  |
| Diagnosis with support from positive and negative history and PE findings | Risk for unintentional firearm injury as children are in home with firearm that they have gained access to. |
| Differential with support from positive and negative history and PE findings |  |
|  |  |
| MANAGEMENT OR DIAGNOSTIC PLAN | Discuss risks for unintentional firearm injuries. Counsel mother on safe firearm storage and prevention of future injuries. |
|  |  |
| PROFESSIONALISM ISSUES OR CHALLENGES: |  |

*Standardized patient - Steven*

Date: September 5, 2020

Primary Case Author: Nikia McFadden

Secondary Case Author: Sarah Stokes

Standardized Patient Educator: Sarah Stokes and Nikia McFadden

Name of Case: Future risk of violence and firearms

Name of educational and or assessment activity: Safe firearm storage counseling

Patient Name: Steven

Chief Complaint: Assault – gunshot wound to right lower extremity

Most likely Diagnosis and Differential with rationale from history and/or physical exam:

Firearm stored unlocked by a patient who was a recent victim of firearm violence

Challenge question:

Domains: Check all that apply

- Professionalism

X Communication and Interpersonal skills

- Medical History
- Physical exam
- Shared Decision Making

X Patient Education

- Clinical Reasoning
- Documentation
- Handoff
- Presentation
- Other:

Type and level of learner: Surgery or Emergency Medicine Resident or Trauma Nurse Practitioner

Case Objectives: please list specific objectives for each of the domains you have checked above:

1. Learner will evaluate patient risks for firearm injury

2. Learner will counsel patient on safe firearm storage

| SETTING: outpatient, in patient, ED, home, nursing home, rehab, group etc. | Inpatient |
| --- | --- |
| PATIENT PROFILE: Information about the “patient” that helps select an SP and helps the learner get an understanding of them as a person. SP will know more information about the patient than learner will ever ask but allows SP to portray a fully developed patient personality. If none of the items below are particulars for the case please write “all may be used.” | |
| Age range | 20-40 years |
| Religious/spiritual background | All may be used |
| Sex (e.g., male, female, intersex, transwoman, transman) | Male |
| Sexual Orientation (e.g., heterosexual, lesbian, gay, bisexual, pansexual, queer, asexual) | Heterosexual |
| Gender expression (e.g., man, woman, gender queer) | Man |
| Race/ethnicity: | Black, non-Hispanic |
| Physical description (e.g., BMI, height range) | All may be used |
| Physical limitations | All may be used |
| Patient appearance (e.g., disheveled, hospital gown, business casual, casual) | Hospital gown |
| Moulage + location (e.g., none, bruises, scars, body piercing, tattoos) | Surgical bandages on left leg |
| Affect (e.g., pleasant, cooperative) | Still shocked about recent violence. Prior to this, no history of violence. Nervous about re-entering the community. |
| Family group (e.g., who is family, who they live with) | Lives with wife and 5-year-old son |
| Education | All may be used |
| Level of health literacy | High |
| Employment, if any - present and past, noting any current stresses | High School Teacher |
| Home/homeless - type of dwelling, number of stories, owned or rented | Lives in owned or rented home alone |
| Financial situation- any current stresses | No acute stresses, able to afford housing, food, clothing, some additional money |
| Insurance Status (e.g., un/under/insured, public/private, HMO/PPO) | Private insurance through employer |
| Habits (i.e., diet, exercise, caffeine, smoking, alcohol, drugs) | Rare alcohol, no drug use |
| Activities (i.e., hobbies, sports, clubs, friends) | Spending time with family and friends |
| Typical day - what is the usual daily routine | Goes to work at the high school, then comes home to spend time with wife and son |

| CASE INFORMATION | |
| --- | --- |
| Chief Concern: What the patient will say when greeted by the student. The patient’s primary reason for seeking medical care often stated in his/own words. | Good morning. My leg is feeling so much better. Can I go home today? |
| Additional Concerns: Other, if any, concerns the patient has today (i.e., symptoms, requests, expectations, etc.) that will become part of set agenda. | If asked ‘Why is firearm stored this way?’: You want to make sure that your kid cannot reach it, so you put it on top of the closet.  Response to being counseled about storing your firearm locked and unloaded: At first you are confused about what your gun has to do with the fact that you were assaulted. However, you are not angry that the provider is bringing up the topic. You politely say that you will consider changing the way you store your gun, but for now you want to be focused on healing your leg. |
|  | |
| THE PATIENT STORY: The SP will be asked to tell their symptom story and the personal and emotion impact for each of their concerns. You will want to write this is the patient voice. The symptom story should be able to answer this question: “Tell me more about [chief concern/additional concern], starting at the beginning and bringing me up to now.”  The personal context should be able to answer questions concerning the broader personal/psychosocial context of symptoms, especially the patient beliefs/attributions.  The emotional context should be able to ask how are you doing with this, how does this make you feel, how has this affected you emotionally? IMPACT: How has this affected your life? How has this been for your family? | I’m still in shock as to how this happened. I was out to dinner with a friend at the local diner. I saw an acquaintance who was sitting a few tables over, so I greeted him. He became upset during our conversation and assaulted me with his firearm. It was all so fast. I still don’t understand why this happened to me. I’m very grateful for your entire team for taking care of me. |
| HISTORY OF PRESENT ILLNESS: Although some of the HPI will be given in the patient’s symptom story, the learners will expand the story during the direct question section. Below describe the detailed history, usually about the chief concern, which the student must develop in order to make a useful assessment of the problem: | |
| Steven is 26-year-old man who was admitted after sustaining a gunshot wound to his left lower extremity. At admission he reported he was shot by an acquaintance while at the local diner. His wound was washed out and he is now preparing for discharge. You are evaluating him prior to discharge and want to discuss safe firearm storage with him.  He lives with his wife and 5-year-old son in a 2-bedroom apartment. His son does have friends over. Either Steven or his wife typically supervises them throughout their playtime, sometimes stepping away to make dinner, but they are always within earshot. He has a single handgun in the home. It is stored in a cardboard box at the top of a closet in the bedroom he shares with his wife. It is stored loaded. He does not think his child knows anything about the gun. His wife knows about the gun but has never interacted with it. He has the gun to defend the home in case someone breaks in. He has taken it to a shooting range once and practiced but have otherwise never used it. | |
| Onset (when; gradual or sudden) | Pain in leg - sudden onset after assault |
| Setting (what was going on or where was patient when symptoms first noticed?) | At local diner |
| Duration (how long) | Prior to arrival |
| Time relationships (frequency, constant or intermittent) | N/A |
| Location | N/A |
| Radiation | N/A |
| Quality | N/A |
| Amount | N/A |
| Aggravated by what | N/A |
| Relieved by what | N/A |
| Associated with what | N/A |
| Attitude (what does the patient think is the problem, and how does he/she feel about it) | You are still shocked that this happened to you. You do not have a history of violence and didn’t know that this acquaintance even had a problem with you. You are nervous about going back into the community. You have some pain, but the medication is helping. |
| Overall course |  |
| REVIEW OF SYSTEMS: Significant positives and negatives | |
| Has firearm (handgun) at home | Denies suicidal or homicidal ideation |
| Firearm is stored loaded | Wife knows where firearm is but doesn’t interact with it. She has no history of suicidal or homicidal ideation. |
| Does not use firearm lock. Stored at top of closet in cardboard box. |  |
| Son has friends over to visit. Patient does not think son knows where firearm is. |  |
|  | |
| Past medical history |  |
| Medication allergies (Name and reaction) | None |
| Environmental allergies (Name and reaction) | None |
| Illnesses | None |
| Vaccinations | Up to date |
| Surgeries | None |
| Accidents/ injuries/ trauma | None prior to current |
| Hospitalization | None prior to current |
|  | |
| Inclusive sexual and reproductive history | |
| Sexual practices  Sexual partners  Protection: Use of safer sex practices  Use of birth control if appropriate  Risk of intimate partner violence | Heterosexual  Wife  Condoms  N/A  No |
| Ob/GYN HISTORY | N/A |
| Medications | Prescription/dose/reason - None  Over the counter/dose/reason - None  Herbs/supplements/dose/reason - None  Other: |
| Immunizations | X Tetanus  X Flu  X Hepatitis  X Pneumovax  X HPV   - Other |
| Tobacco products:   - Cigarettes - Cigar - Pipe - Chew - E-cigarettes | X Never   - Past- year started/year quit - Current   - Quantity   - # of years |
| Alcohol  X Beer   - Wine - Liquor - Other | - Never - Past- year started/year quit   X Current   - - Rare use – 1 drink every couple of months   - # of years – 5 years |
| Drugs   - Weed - Cocaine - Heroin - Meth - Other - IV - Inhalants - Other | X Never   - Past- year started/year quit - Current   - Quantity - # of years |
| Diet (describe) | Any |
| Exercise (describe) | Goes running 1x per week |
| List any other important social history or information important to this case |  |
| Family history |  |
| Mother, Father, Siblings, Grandparents, and other significant findings. | None |
|  |  |
| Physical Exam- List exam maneuvers expected for this case and any abnormal findings that SP will simulate. (tenderness, hyper-hypo reflex, rebound, weakness etc. )  No physical exam expected. | |
| PHYSICAL EXAM FINDINGS |  |
| 1. Written in layman’s terms |  |
| 1. General appearance- affect, appearance, position of patient at opening (i.e. sitting, laying down, holding abdomen etc.) |  |
| 1. Vital signs |  |
| 1. Specific findings and affect |  |
| 1. Response to certain physical movements |  |
|  |  |
| DIAGNOSIS AND DIFFERENTIAL |  |
| Diagnosis with support from positive and negative history and PE findings | Patient is a victim of firearm violence who owns a firearm that is stored unlocked and loaded. In addition, the patient lives at home with his son who is 5 years old. Due to this recent history, he is at increased risk of being involved in violence in the future. In addition, there is risk of unintentional injury to his son. |
| Differential with support from positive and negative history and PE findings |  |
|  |  |
| MANAGEMENT OR DIAGNOSTIC PLAN | Counsel patient on safe firearm storage, describing why the patient may be at higher risk. Describe options for safe firearm storage. |
|  |  |
| PROFESSIONALISM ISSUES OR CHALLENGES: |  |
