# Supplementary material for: Firearm Safety Counseling for Patients: An Interactive Curriculum for Trauma Providers
Source: MedEdPORTAL. 2022 May 10;18:11237. doi: 10.15766/mep_2374-8265.11237 (PMC9085984; doi:10.15766/mep_2374-8265.11237)
Supplement: Supplementary file 1 — Safe Firearm Storage.pptxStandardized Patient Cases.docxPresentation of Standardized Patient Cases.docxPre- and Postsurveys.docx [file mep_2374-8265.11237-s001.zip › C. Presentation of Standardized Patient Cases.docx]

**Appendix C.**  Presentation of standardized patient cases to participants

**Educational Objectives**

By the end of this curriculum learners will be able to:

1. Describe the status of firearm injuries in the United States
2. Identify patients at risk of firearm injury and violence
3. Describe and analyze options for safe storage of a firearm and identify locally available free options
4. Apply skills to discuss firearm storage with patients and provide education on safe storage options

The goal of this simulation is to practice the safe firearm storage counseling you learned in the lecture. Your group will meet with three standardized patients who sustained a traumatic injury themselves or are the parent of a child who sustained a traumatic injury.

Your goal in each conversation is to evaluate the risk of future firearm injury, assess the patient’s/parent’s current firearm storage practices and provide counseling about safe firearm storage. Each conversation will last about 7 minutes. At the end of the conversation the standardized patient will provide you with feedback. Members of the group who are not participating in the discussion should also provide feedback on the encounter. When providing feedback to your peers consider:

- What do you think the learner did well and what do you think they could have improved?
- Do you think the learner clearly conveyed how to safely store firearms?
- How did the learner engage the standardized patient in the firearm safe storage discussion?
- What similar experiences have you had and have you been able to previously discuss firearm storage with patients?

Case 1: Richard

Richard is a 60-year-old man was admitted after attempting suicide by cutting his wrists. His injuries were repaired. He was evaluated in the hospital by psychiatry, and was deemed to be not at acute risk of suicide. You are evaluating him prior to discharge and want to discuss safe firearm storage with him.

Case 2: Anna

Anna is the mother of a 6-year-old boy, Max, who was admitted after he accidentally shot himself while playing at home. At admission you found out that Max had been playing alone, found his father’s gun and accidentally shot himself in the arm. He was taken to the operating room and had the wound washed out. He is recovering well and preparing for discharge. The family has been evaluated by social work, there is no concern for abuse or neglect. You are evaluating Max prior to discharge and want to discuss safe firearm storage with his mother.

Case 3: Steven

Steven is 26 year old man who was admitted after sustaining a gunshot wound to his left lower extremity. At admission he reported he was shot by an acquaintance. His wound was washed out and he is now preparing for discharge. You are evaluating him prior to discharge and want to discuss safe firearm storage with him.
