# Supplementary material for: Firearm Safety Counseling for Patients: An Interactive Curriculum for Trauma Providers
Source: MedEdPORTAL. 2022 May 10;18:11237. doi: 10.15766/mep_2374-8265.11237 (PMC9085984; doi:10.15766/mep_2374-8265.11237)
Supplement: Supplementary file 1 — Safe Firearm Storage.pptxStandardized Patient Cases.docxPresentation of Standardized Patient Cases.docxPre- and Postsurveys.docx [file mep_2374-8265.11237-s001.zip › D. Pre- and Postsurveys.docx]

**Appendix D. Firearm Safety Education Pre- and Postdidactic Session Survey**

What is your age?*

­__________

What is your gender?*

1. Male
2. Female
3. Non-binary
4. Other
5. Prefer not to answer

What is your position?*

1. Emergency Medicine Resident
2. Surgery Resident
3. Nurse Practitioner

If you are a resident, what is your post graduate year?* (If you are a surgery research resident, select your clinical year as PGY-3)

1. PGY-1
2. PGY-2
3. PGY-3
4. PGY-4
5. PGY-5

If you are a Nurse Practitioner, how many years have you been working in trauma?*

__________

Did you grow up with a firearm in your household?*

1. Yes
2. No
3. Not sure

|  | Strongly agree | Agree | Neither agree nor disagree | Disagree | Strongly Disagree |
| --- | --- | --- | --- | --- | --- |
| I know what types of firearm locks exist and how to use them |  |  |  |  |  |
| I know the optimal way to store a firearm when it is not in use |  |  |  |  |  |
| I have ready access to information that can help patients and families learn more information about safe firearm storage |  |  |  |  |  |
| Compared to other health risks, firearms pose a very significant threat |  |  |  |  |  |
| Physicians and nurse practitioners have a responsibility to counsel patients on firearm safety |  |  |  |  |  |
| Trauma providers should advocate for legislation to reduce the risk of firearm injury |  |  |  |  |  |
| I will offend a patient or parent of a patient if I discuss firearm safety with them |  |  |  |  |  |
| I would be offended if my healthcare provider discussed firearm safety with me |  |  |  |  |  |
| Patients will change their actions around firearm storage based on physician or nurse practitioner discussion |  |  |  |  |  |
| When I see a trauma patient in the hospital or in the emergency department, there is enough time to talk about firearm safety |  |  |  |  |  |
| Children are safer with a firearm in the home |  |  |  |  |  |
| Adults are safer with a firearm in the home |  |  |  |  |  |
| I am confident that I can effectively counsel patients and parents of patients about safe firearm storage |  |  |  |  |  |
| I am confident that I can effectively counsel a patient at risk of suicide about options for firearm disposal or storage outside their home |  |  |  |  |  |
| I have had adequate training to counsel patients and their families about safe firearm storage |  |  |  |  |  |

| In the past 3 years I have taken care of a child (<18 years) who* … | | |
| --- | --- | --- |
|  | Yes | No |
| … was injured by a firearm |  |  |
| … died after a firearm injury |  |  |
| … died by suicide with a firearm |  |  |

| In the past 3 years I have taken care of an adult (>18 years) who* … | | |
| --- | --- | --- |
|  | Yes | No |
| … was injured by a firearm |  |  |
| … died after a firearm injury |  |  |
| … died by suicide with a firearm |  |  |

| In the past year I have heard about or discussed a case of* … | | |
| --- | --- | --- |
|  | Yes | No |
| … a child who was injured or died after a firearm injury |  |  |
| … an adult who was injured or died after a firearm injury |  |  |

*These questions were included only in the pre-session survey
