# Supplementary material for: Firearm Safety Counseling for Patients: An Interactive Curriculum for Trauma Providers
Source: MedEdPORTAL. 2022 May 10;18:11237. doi: 10.15766/mep_2374-8265.11237 (PMC9085984; doi:10.15766/mep_2374-8265.11237)
Supplement: Supplementary file 1 — Safe Firearm Storage.pptxStandardized Patient Cases.docxPresentation of Standardized Patient Cases.docxPre- and Postsurveys.docx [file mep_2374-8265.11237-s001.zip › Educational_Summary_Report_11237.pdf]

MedEdPORTAL® The AAMC Journal of Teaching and Learning Resources

Original Publication Open Access

Firearm Safety Counseling for Patients: An Interactive Curriculum for Trauma Providers

Sarah C. Stokes, MD*, Nikia R. McFadden, MD, Edgardo S. Salcedo, MD, Alana L. Beres, MD, MPH

*Corresponding Author: scstokes@ucdavis.edu

Abstract

Introduction: Firearm injuries are a major public health concern. Safe firearm storage is recommended by multiple medical organizations. However, rates of firearm safety counseling are particularly low among trauma providers. Educational initiatives for other provider groups have proven to be effective. We hypothesized that educating trauma providers to offer safety counseling would be similarly effective. **Methods:** We developed a didactic session around safe firearm storage counseling for trauma providers consisting of a lecture followed by an interactive session with standardized patients. Session participants completed pre- and postsurveys evaluating their knowledge about firearm storage, self-efficacy in providing firearm storage counseling, and attitudes towards firearm safety. We compared differences between pre- and postsurvey data using chi-square tests.**Results:** The didactic session was delivered to target trauma providers: three trauma nurse practitioners, 42 general surgery residents, and 26 emergency medicine residents. After the session, participants were more likely to know the optimal way to safely store a firearm and to be confident in effectively counseling patients about safe firearm storage. Learners were not more likely to believe that providers have a responsibility to counsel patients on firearm safety. **Discussion:** A didactic session on safe firearm storage counseling was associated with increased rates of knowledge and self-efficacy. The session did not change attitudes among trauma providers, although, prior to the session, most providers already believed they had a responsibility to counsel patients on safe firearm storage. Similar curricula should be piloted at other trauma centers.

Keywords:
Firearms, Advocacy, Emergency Medicine, General Surgery, Standardized Patient

Educational Objectives

By the end of this curriculum, learners will be able to:

1. Describe the status of firearm injuries in the United States.
2. Identify patients at risk of firearm injury and violence.
3. Describe and analyze options for safe storage of a firearm and identify locally available free options.
4. Apply skills to discuss firearm storage with patients and provide education on safe storage options.

Introduction

Firearm injuries are a continued major public health concern.[1] In the United States, between 2009 and 2017, an average of 120,232 people were injured by firearms both intentionally and unintentionally annually.[1] Storing firearms loaded and unlocked has been demonstrated to be associated with increased risk of suicide and unintentional injury.[2,3] Safe firearm storage, defined as storing firearms locked and unloaded, is recommended by multiple medical organizations, including the American College of Surgeons, American Pediatric Surgical Association, American College of Physicians, and Eastern Association for the Surgery of Trauma.[4-7]

Physician counseling can lead to safe storage of firearms by patients.[8,9] Safe storage counseling may be particularly important after traumatic injuries, as patients who have experienced a traumatic event are at increased risk of future traumatic injuries and suicide and are more likely to acquire a firearm in the future.[10,11] However, many physicians do not counsel patients on safe firearm storage, and firearm safety counseling is particularly rare among trauma providers.[12,13]

Educational initiatives for pediatric residents have increased both rates of counseling by residents and resident comfort with counseling.[14-17] Unlike pediatric residents, trauma providers

Citation:
Stokes SC, McFadden NR, Salcedo ES, Beres AL. Firearm safety counseling for patients: an interactive curriculum for trauma providers. *MedEdPORTAL*. 2022;18:11237.
https://doi.org/10.15766/mep_2374-8265.11237

Copyright © 2022 Stokes et al. This is an open-access publication distributed under the terms of the Creative Commons Attribution license.

typically do not counsel patients on high-risk behaviors.[18] Additionally, while pediatric residents meet patients in a clinic setting, trauma providers meet patients in an acute setting. Therefore, training for counseling must be adapted both to less experienced learners and to the acute setting. We hypothesized that if trauma providers were given education on how to offer safe firearm storage counseling, they could achieve similar results to those of pediatric residents. We developed a didactic session to educate trauma providers—including general surgery (GS) residents, emergency medicine (EM) residents, and trauma nurse practitioners (NPs)—on safe firearm storage counseling. We designed the session to include a lecture followed by a practice session with standardized patients. Including lectures reviewing the appropriate material just prior to standardized patient sessions has been shown to increase learner enjoyment and the value of the standardized patient session.[19] Involvement of standardized patients in medical education has been demonstrated to improve learner confidence,[20] which we viewed as a critical component of our educational goals. We divided learners into small groups to complete the standardized patient sessions in order to maximize the number of learners able to participate in a standardized patient scenario. Through our didactic session, we aimed to improve learner self-efficacy and knowledge about safe firearm storage counseling.

Methods

Settings and Learners

We developed a didactic session on safe firearm storage counseling at UC Davis Health (UCDH), a level 1 pediatric and adult trauma center in Sacramento, California, with dedicated GS and EM residency programs. At our institution, GS and EM residents both have clinical exposure to firearm injuries. The trauma department admits approximately 4,000 patients per year and sees a significant amount of firearm trauma. On presentation to the emergency department, every major trauma is comanaged by the emergency and trauma surgery departments. Therefore, both residency groups are heavily involved in taking care of patients who have experienced firearm trauma. GS residents spend at least 2 months per postgraduate year on trauma service. At the time of this project, trauma providers at our institution had received no formal education in safe firearm storage counseling. The project was approved with a waiver for informed consent by the Institutional Review Board at UCDH (IRB no. 1606318). The education sessions were provided for trauma NPs, GS residents, and EM residents. These didactic sessions were provided as stand-alone sessions during mandatory education time for GS and EM residents as well as optional education time for trauma NPs.

Curriculum Design and Preparation

Our didactic session on safe firearm storage for trauma providers consisted of a short lecture followed by an interactive session with standardized patients. The session was developed by reviewing existing safe firearm storage curricula,[17] literature regarding counseling patients on firearm safety,[21,22] and literature detailing the current state of firearm violence in the United States.[5,23,24] We collaborated with the BulletPoints Project,[25] a team of clinicians, researchers, and public health experts that provides clinical tools for firearm injury prevention. One tool they have developed is an online curriculum to teach providers about firearm injury prevention. Their website includes resources for providers on prevention of suicide, unintentional firearm injury, recurrent injury, intimate partner violence, and community violence.

Our team also met with local law enforcement to discuss safe firearm storage, local firearm laws, and local firearm safety resources. A preliminary version of the didactic session was reviewed and revised by a multidisciplinary team composed of adult trauma surgeons, pediatric trauma surgeons, and GS resident physicians.

Two of the authors (Sarah C. Stokes and Nikia R. McFadden) led three safe firearm storage education sessions—one for each group—for trauma NPs, GS residents, and EM residents. Didactic sessions lasted approximately 60 minutes and occurred either in person or over Zoom, due to social distancing measures necessitated by the COVID-19 pandemic. In the first 30 minutes of the session, we presented a lecture (Appendix A) that covered firearm injury statistics in the US with a specific focus on unintentional injuries and suicides, the association of these injuries with unsafely stored firearms, the potential benefit of provider safe firearm storage counseling, and local and national laws related to firearms and firearm safety discussions. The lecture also included basic firearm knowledge and discussed various safety and storage devices—including average prices for each and where to obtain them for free. Next, we discussed how to legally surrender firearms, resources for more information about firearm safety counseling, and patient resources for more information about firearm safety. Finally, we reviewed strategies for counseling patients and families about safe firearm storage.

Following the lecture, we divided learners into groups of five to six for interactive sessions with standardized patients to apply the learners' newly attained firearm safety knowledge in a clinical practice setting. Standardized patients were recruited through the University of California, Davis, Clinical Education Resource Center. We met with the standardized patients in

Copyright © 2022 Stokes et al. This is an open-access publication distributed under the terms of the Creative Commons Attribution license.

advance to review and practice each scenario (Appendix B). The standardized patients were also given the opportunity to provide feedback and ask for additional clarifying information. They interacted with the learning groups for a total of 30 minutes, completing three case scenarios. Participant introductions to the cases (Appendix C) were also provided. We met with the standardized patients between didactic sessions to review any questions or issues that arose.

During each simulated clinical scenario, a single provider participant practiced safe firearm storage counseling with a standardized patient for 7 minutes and then received feedback for 3 minutes. Providers who did not interact with the standardized patient observed each counseling session and were able to ask questions during the feedback section. We instructed the standardized patients to provide feedback to participants on the learner's clarity of counseling and ability to provide nonjudgmental counseling. We asked learners to discuss the following questions during the debrief after the standardized patient scenarios:

- What do you think the learner did well, and what do you think they could have improved upon?
- Do you think the learner clearly conveyed how to safely store firearms?
- How did the learner engage the standardized patient in the firearm safe storage discussion?
- What similar experiences have you had, and have you been able to previously discuss firearm storage with patients?

Learner Assessment and Session Evaluation

Learners in the session completed a survey before and after the didactic session that evaluated prior experience with firearms, knowledge related to safe firearm storage, self-efficacy (a person's belief that they have the ability to perform a task[26]) in providing firearm storage counseling, and attitudes towards firearms (Appendix D). We developed pre- and postdidactic surveys based on barriers to firearm safety counseling reported in the literature[15,27,28] and safe firearm storage counseling educational surveys identified in the literature.[27,29,30] Preliminary versions of the pre- and postdidactic surveys were reviewed and revised by an interdisciplinary team that included GS residents, trauma surgeons, and pediatric surgeons. The survey was pilot tested with GS residents and revised for clarity.

In the predidactic survey, we asked about the following:

- Learner personal experience with firearms.
- Learner clinical experience caring for patients with firearm injuries.
- Learner knowledge about firearm storage.
- Attitudes and beliefs about the role of trauma providers in providing safe firearm storage counseling.
- Self-efficacy.

In the postdidactic survey, we asked about the following:

- Learner knowledge about firearm storage.
- Attitudes and beliefs about the role of trauma providers in providing safe firearm storage counseling.
- Self-efficacy.

Questions were based on a 5-point Likert scale (1 = *strongly agree,* 5 = *strongly disagree*). We distributed the surveys and collected data via Qualtrics software. Surveys were optional and anonymous, and no identifying data were collected from learners. We did not provide any financial incentives for participation in the project.

Primary outcomes of interest were knowledge of safe firearm storage and firearm safety counseling self-efficacy. Five-point Likert-scale survey scores were transformed into binary variables as follows: The variable *agree* was formed from scores of 1 (*strongly agree*) and 2 (*somewhat agree*), and the variable *unsure or does not agree* was formed from scores of 3 (*unsure*), 4 (*somewhat disagree*), and 5 (*strongly disagree*). Demographic and survey data were summarized for the project population using counts and percentages. Chi-square tests were used to compare differences between pre- and postsurvey data. We used logistic regression and chi-squares to test for associations between various survey responses on the presurvey. SAS 9.4 (SAS Institute) was used for all statistical analyses, with statistical significance at $p < .05$.

Results

We led the safe firearm storage didactic sessions in September and October 2020. We delivered the didactic session to three learners at the NP session, 42 learners at the GS resident session, and 26 learners at the EM resident session (Table 1). Everyone ($N = 71$) who attended the didactic session completed the presurvey, and 26 learners (37%) completed the postsurvey. Full demographic details for learners are listed in Table 1. Most learners reported prior clinical experience with children and adults who were injured or died from a firearm (Table 2).

Knowledge

On the predidactic survey, 46% ($n = 32$) of learners correctly identified what types of firearm locks exist and how to use them,

Copyright © 2022 Stokes et al. This is an open-access publication distributed under the terms of the Creative Commons Attribution license.

Table 1. Education Session Participant Demographics

Demographic	No. (%)
Position (presurvey)[a]	
Emergency medicine resident	26 (37)
Surgery resident	42 (59)
Trauma nurse practitioner	3 (4)
Position (postsurvey)[b]	
Emergency medicine resident	11 (42)
Surgery resident	13 (50)
Trauma nurse practitioner	2 (8)
Postgraduate year[c]	
PGY 1	23 (34)
PGY 2	13 (19)
PGY 3	19 (28)
PGY 4	7 (10)
PGY 5	6 (9)
Grew up in a household with a firearm	
Yes	35 (49)
No	36 (51)
	***Mdn* (Range)**
Years working in trauma[d]	12 (5-12)

[a]Seventy-one participants completed the presurvey.
[b]Twenty-six participants completed the postsurvey.
[c]Emergency medicine and surgery residents only (n = 68). Of note, emergency medicine residents comprise PGY 1-PGY 3 only.
[d]Nurse practitioners only (n = 3).

61% ($n = 43$) knew the optimal way to store a firearm when it is not in use, and 16% ($n = 11$) knew where to find information that could help patients and families learn more about safe firearm storage (Table 3). There was a significant association between growing up with a gun in the household and knowing what types of gun locks exist and how to use them ($p < .001$). After the didactic session, learners were more likely to know what types of gun locks exist and how to use them ($n = 26$, 100%; relative risk [RR] = 2.2; 95% CI, 1.7-2.8), were more likely to know the optimal way to store a firearm when it is not in use ($n = 26$, 100%; RR = 1.6; 95% CI, 1.4-2.0), and were more likely to know where to access information to help patients and families learn more about safe firearm storage ($n = 18$, 69%; RR = 4.4; 95% CI, 2.4-8.0; Table 3).

Attitudes and Beliefs

On the presurvey, 72% ($n = 51$) of learners believed that firearms pose a significant threat compared to other health risks, 77% ($n = 55$) believed that physicians and NPs have a responsibility to counsel patients on firearm safety, and 80% ($n = 57$) believed that trauma providers should advocate for legislation to reduce the risk of firearm injury (Table 3). After the didactic session, learners were not more likely to believe that firearms pose a significant health threat compared to other health risks ($n =$ 22, 85%; RR = 1.2; 95% CI, 0.9-1.5), were not more likely to believe that physicians and NPs have a responsibility to counsel patients on firearm safety ($n = 22$, 85%; RR = 1.1; 95% CI, 0.9-1.3), and were not more likely to believe that trauma providers should advocate for legislation to reduce the risk of firearm injury ($n = 20$, 77%; RR = 1.0; 95% CI, 0.8-1.2; Table 3).

On the predidactic survey, 53% ($n = 38$) of providers believed that patients would change their actions around firearm storage based on physician or NP discussion, and 18% ($n = 13$) believed that there is enough time to discuss firearm safety with their trauma patients. After the didactic session, learners were more likely to believe that patients would change their actions around firearm storage based on physician or NP discussion ($n = 22$, 85%; RR = 1.6; 95% CI, 1.2-2.1) and were more likely to believe that there is enough time to discuss firearm safety with their trauma patients ($n = 11$, 42%; RR = 2.3; 95% CI, 1.2-4.5; Table 3).

On the predidactic survey, 8% of providers ($n = 6$) believed that children are safer with a firearm in the home, and 24% ($n = 17$) of providers believed that adults are safer with a firearm in the home (Table 3). After the didactic session, providers were not less likely to believe that children are safer with a firearm in the home ($n = 1$, 5%; RR = 0.5; 95% CI, 0.1-3.6) and were not less likely to believe that adults are safer with a firearm in the home ($n = 5$, 19%; RR = 0.8; 95% CI, 0.3-2.0; Table 3).

Table 2. Participant Clinical Experiences

Statement	No. (%)		
	**Yes**	**No**	**Unsure**
In the past 3 years, I have taken care of:			
A child who was injured by a firearm.[a]	61 (90)	7 (10)	0 (0)
A child who died from a firearm injury.[a]	33 (49)	25 (37)	10 (15)
A child who died by suicide using a firearm.[b]	9 (13)	49 (73)	9 (13)
An adult who was injured by a firearm.[a]	66 (97)	2 (3)	0 (0)
An adult who died from a firearm injury.[a]	62 (91)	5 (7)	1 (1)
An adult who died by suicide using a firearm.[b]	34 (51)	20 (30)	13 (19)
In the past year, I have heard about or discussed a case of:			
A child who was injured or died after a firearm injury.[a]	59 (87)	8 (12)	1 (1)
An adult who was injured or died after a firearm injury.[c]	68 (99)	1 (1)	0 (0)

[a]Sixty-eight participants responded to the statement.
[b]Sixty-seven participants responded to the statement.
[c]Sixty-nine participants responded to the statement.

Copyright © 2022 Stokes et al. This is an open-access publication distributed under the terms of the Creative Commons Attribution license.

Table 3. Pre- and Postsurvey Learner Knowledge, Attitudes and Beliefs, and Self-Efficacy

Statement	No. (%) Agreeing		RR (95% CI)	*P*
	Presurvey (*N* = 71)	Postsurvey (*N* = 26)		
Knowledge				
I know what types of firearm locks exist and how to use them.	32[a] (46)	26 (100)	2.2 (1.7-2.8)	<.001
I know the optimal way to store a firearm when it is not in use.	43[a] (61)	26 (100)	1.6 (1.4-2.0)	.002
I have ready access to information that can help patients and families learn more information about safe firearm storage.	11[a] (16)	18 (69)	4.4 (2.4-8.0)	<.001
Attitude and beliefs				
Compared to other health risks, firearms pose a very significant threat.	51 (72)	22 (85)	1.2 (0.9-1.5)	.20
Physicians and nurse practitioners have a responsibility to counsel patients on firearm safety.	55 (77)	22 (85)	1.1 (0.9-1.3)	.40
Trauma providers should advocate for legislation to reduce the risk of firearm injury.	57 (80)	20 (77)	1.0 (0.8-1.2)	.70
I will offend a patient or parent of a patient if I discuss firearm safety with them.	14 (20)	2 (8)	0.4 (0.1-1.6)	.20
I would be offended if my health care provider discussed firearm safety with me.	3 (4)	0 (0)	NA	.30
Patients will change their actions around firearm storage based on physician or nurse practitioner discussion.	38 (53)	22 (85)	1.6 (1.2-2.1)	.005
When I see a trauma patient in the hospital or in the emergency department, there is enough time to talk about firearm safety.	13 (18)	11 (42)	2.3 (1.2-4.5)	.02
Children are safer with a firearm in the home.	6 (8)	1 (5)	0.5 (0.1-3.6)	.40
Adults are safer with a firearm in the home.	17 (24)	5 (19)	0.8 (0.3-2.0)	.60
Self-efficacy				
I am confident that I can effectively counsel patients and parents of patients about safe firearm storage.	26[a] (37)	23 (88)	2.4 (1.7-3.3)	<.001
I am confident that I can effectively counsel a patient at risk of suicide about options for firearm disposal or storage outside their home.	14[a] (20)	24 (92)	4.6 (2.9-7.5)	<.001
I have had adequate training to counsel patients and their families about safe firearm storage.	10[a] (14)	25 (96)	6.7 (3.8-12.0)	<.001

Abbreviation: RR, relative risk.
[a]Seventy participants responded to this presurvey statement.

Self-Efficacy

On the predidactic survey, 37% ($n = 26$) of learners were confident that they could effectively counsel on safe firearm storage, 20% ($n = 14$) were confident that they could effectively counsel a patient at risk of suicide about options for firearm disposal or storage outside their home, and 14% ($n = 10$) felt that they have had adequate training to counsel patients and their families about safe firearm storage (Table 3). After the didactic session, learners were more likely to be confident that they could effectively counsel patients and parents of patients about safe firearm storage ($n = 23$, 88%; RR = 2.4; 95% CI, 1.7-3.3), were more likely to be confident that they could effectively counsel a patient at risk of suicide about options for firearm disposal or storage outside their home ($n = 24$, 92%; RR = 4.6; 95% CI, 2.9-7.5), and were more likely to feel that they have had adequate training to counsel patients and their families about safe firearm storage ($n = 25$, 96%; RR = 6.7; 95% CI, 3.8-12.0; Table 3).

On the predidactic survey there was a significant association between those who felt confident that they could effectively counsel patients and parents of patients about safe firearm storage and learners who grew up with a firearm in the household ($p = .01$), as well as those who felt they had adequate training to counsel patients and their families about safe firearm storage ($p < .001$). There was no association between PGY level and confidence counseling patients and parents of patients about safe firearm storage (PGY 5 vs. PGY 1, $p = 1.0$).

Discussion

We developed a didactic session, which included a lecture and an interactive standardized patient session, on firearm storage counseling for trauma providers using nationally recognized materials. The lecture portion provided learners with data on the current state of firearm injuries in the United States, clear information on how firearms can be stored safely, and guidance on providing effective counseling in an acute care setting. The standardized patient portion was an interactive session that engaged learners in actively applying newly acquired knowledge in counseling patients. We found that this didactic session improved knowledge and self-efficacy in safe firearm storage counseling for trauma providers. Prior to the didactic session, the majority of learners believed that firearms do pose a significant health risk and that providers should be involved in patient education and advocacy, and therefore, the didactic session did not significantly change those beliefs.

Provider self-efficacy in counseling is closely related to the likelihood that providers will counsel patients, and a lack of confidence in discussing firearm safety is frequently cited by providers as a barrier to providing counseling.[15,27,28] We found

Copyright © 2022 Stokes et al. This is an open-access publication distributed under the terms of the Creative Commons Attribution license.

an increase in participants' confidence in their ability to effectively counsel patients on safe firearm storage and to counsel patients at risk of suicide. Prior studies of educational initiatives in pediatric residents have demonstrated an improvement in self-efficacy with training.[15,17] Evaluating firearm safety counseling in provider populations other than pediatricians is important, as pediatricians provide preventative care counseling more frequently than physicians in other specialties.[31] Having prior experience counseling patients on preventative care may increase the comfort that pediatric residents have in providing safe firearm storage counseling. In contrast, trauma providers rarely provide counseling to patients on high-risk behaviors.[13,18,32] The lack of experience with providing preventative counseling means that a curriculum for trauma providers should be tailored to include information on how to approach preventative counseling as well as practice with standardized patients so learners can develop new skills. In our didactic session, we provided information on how to approach conversations about safe storage and allowed learners the opportunity to practice scenarios with standardized patients, which we believe contributed to success in increasing self-efficacy.

Another noted barrier to safe storage counseling is a knowledge deficit. Similar to the baseline results in our survey, low rates of knowledge regarding safe firearm storage have also been reported amongst pediatric residents.[15,16] Half of the learners in our didactic sessions did not grow up with a firearm in the household, and this was closely correlated with a lack of knowledge of safe firearm storage and firearm locking devices. A significant portion of our lecture was dedicated to providing clear and specific information on methods of safe firearm storage. Consultation with local law enforcement was useful in ensuring the accuracy of this information.

At baseline, a high percentage of learners believed that physicians and NPs have a responsibility to counsel patients on firearm safety (77%) and that firearms pose a significant health threat (72%). These attitudes did not significantly change with the didactic session, although this was not unexpected given such a high percentage who believed prior to the session in the responsibility of providers to counsel. Having experience caring for a patient who has sustained a firearm injury increases the likelihood that providers will counsel on firearm safety and will think that firearms are a health risk.[33] Therefore, the experience of learners, many of whom reported recently caring for adults and children injured by firearms, may have impacted their attitudes and beliefs regarding the importance of firearm safe storage. Learners' attitudes likely impacted their desire to acquire knowledge about firearm safety and their willingness to provide counseling to these patients.

In leading these sessions, we were pleased to see active learner engagement with standardized patients, robust discussion from learners after patient scenarios, and thoughtful questions from learners about how to approach patients and what information to provide them. Learners were very involved in the discussions after the standardized patient scenarios and were able to include their prior experience with patients who had sustained firearm injuries. Additionally, learners who owned or had used firearms were able to provide additional insight into firearm storage habits.

We identified some areas for improvement in our curriculum. Learners would benefit from a longer time to practice with standardized patients. In our session, each patient scenario was limited to 7 minutes, but learners frequently did not complete counseling patients in this time frame. As mentioned, the cases were often followed by robust discussion from learners on how their clinical practice could be impacted. These discussions sometimes had to be cut short due to time constraints. In all the scenarios, we provided the standardized patients with stored firearms in a nonoptimal method; however, most firearm owners do store their firearms safely. It would have been beneficial to include a scenario in which the standardized patient practiced safe storage, as learners may frequently encounter this situation in clinical practice.

Limitations

This project had several limitations. First, it was conducted at an urban academic level 1 trauma center that frequently cares for patients with firearm injuries. Therefore, the results may not be applicable to trauma providers in other practice settings. At our center, we have a standardized protocol for providing counseling on alcohol use to trauma patients, which may have made our learners more likely to see a benefit in providing counseling on firearms. Additionally, most learners at our center believed that firearms pose a significant health threat. At programs where trainees hold different beliefs or where trainees encounter fewer patients who have been injured by firearms, there may be a need for increased education on firearm injuries and deaths.

Second, since a significant proportion of learners did not complete the postsurvey (63%), there may be bias regarding those who did that could have impacted our results. We suspect that learners who responded to the postdidactic survey were those who found the session particularly educational; therefore, our results may overestimate the session's effectiveness.

Copyright © 2022 Stokes et al. This is an open-access publication distributed under the terms of the Creative Commons Attribution license.

Interviewing learners after the didactic session might have provided us with additional useful feedback on the benefits of the session. Additionally, the low sample size may have limited our ability to identify significant results.

Third, due to low turnout at the NP didactic session, we mainly focused on resident learners in this curriculum. We acknowledge that there is a benefit to expanding the curriculum to other learners, in particular to nursing staff and social workers. Lastly, adaptation of the curriculum as is requires use of standardized patients—a resource to which not everyone has access. However, we believe that other programs can easily adapt our scenarios if they do not have access to standardized patients by using role-playing. Members of each team can swap roles so that every learner has the opportunity to gain experience in counseling.

Despite these limitations, we present here the first safe firearm storage curriculum for trauma providers that incorporates standardized patients. Trauma providers frequently care for adults and children who are injured by firearms; therefore, the ability to counsel patients on safe firearm storage is critical. To do so, learners must acquire knowledge about how to safely store firearms and develop skills to navigate discussions on this contentious issue. We believe that this curriculum prepares learners to have these important conversations.

Conclusions

The educational didactic on safe firearm storage counseling we developed for trauma providers was associated with increased rates of self-efficacy related to firearm safety counseling and knowledge about safe practices amongst participants. Similar curricula should be piloted at other trauma centers. Our team's ultimate goal is to increase the number of firearm safety discussions between trauma providers and patients. Ongoing research at our institution seeks to evaluate whether the implementation of our firearm safety counseling education leads to an increase in rates of counseling for our trauma patients.

Appendices

A. Safe Firearm Storage.pptx

B. Standardized Patient Cases.docx

C. Presentation of Standardized Patient Cases.docx

D. Pre- and Postsurveys.docx

All appendices are peer reviewed as integral parts of the Original Publication.

Sarah C. Stokes, MD: Resident, Department of Surgery, University of California, Davis, School of Medicine; ORCID: https://orcid.org/0000-0001-7227-1326

Nikia R. McFadden, MD: Resident, Department of Surgery, University of California, Davis, School of Medicine

Edgardo S. Salcedo, MD: Professor, Department of Surgery, University of California, Davis, School of Medicine

Alana L. Beres, MD, MPH: Associate Professor, Department of Surgery, University of California, Davis, School of Medicine

Acknowledgments

Dr. Sarah C. Stokes and Dr. Nikia R. McFadden contributed equally to this work and are co-primary authors.

The authors would like to thank the University of California, Davis, Clinical Education Resource Center for assistance in standardized patient recruitment.

Disclosures

None to report.

Funding/Support

This research was supported by the grant program of the University of California Firearm Violence Research Center at the University of California, Davis. This work was also supported by a training grant from the National Center for Advancing Translational Sciences, National Institutes of Health (UL1 TR001860) for author Nikia R. McFadden. The funding agencies had no involvement in the study design; the collection, analysis, and interpretation of data; the writing of the report; or the decision to submit the report for publication.

Ethical Approval

The UC Davis Health Institutional Review Board approved this project.

References

1. Kaufman EJ, Wiebe DJ, Xiong RA, Morrison CN, Seamon MJ, Delgado MK. Epidemiologic trends in fatal and nonfatal firearm injuries in the US, 2009-2017. *JAMA Intern Med*. 2021;181(2): 237-244. https://doi.org/10.1001/jamainternmed.2020.6696
2. Grossman DC, Mueller BA, Riedy C, et al. Gun storage practices and risk of youth suicide and unintentional firearm injuries. *JAMA*. 2005;293(6):707-714. https://doi.org/10.1001/jama.293.6.707
3. Shenassa ED, Rogers ML, Spalding KL, Roberts MB. Safer storage of firearms at home and risk of suicide: a study of protective factors in a nationally representative sample. *J Epidemiol Community Health*. 2004;58(10):841-848. https://doi.org/10.1136/jech.2003.017343
4. Violano P, Bonne S, Duncan T, et al. Prevention of firearm injuries with gun safety devices and safe storage: an Eastern Association for the Surgery of Trauma systematic review. *J Trauma Acute Care Surg*. 2018;84(6):1003-1011. https://doi.org/10.1097/TA.0000000000001879

Copyright © 2022 Stokes et al. This is an open-access publication distributed under the terms of the Creative Commons Attribution license.

5. Petty JK, Henry MCW, Nance ML, Ford HR; APSA Board of Governors. Firearm injuries and children: position statement of the American Pediatric Surgical Association. *J Pediatr Surg*. 2019;54(7):1269-1276. https://doi.org/10.1016/j.jpedsurg.2019.03.001
6. Butkus R, Doherty R, Bornstein SS; Health and Public Policy Committee of the American College of Physicians. Reducing firearm injuries and deaths in the United States: a position paper from the American College of Physicians. *Ann Intern Med*. 2018;169(10):704-707. https://doi.org/10.7326/M18-1530
7. Talley CL, Campbell BT, Jenkins DH, et al. Recommendations from the American College of Surgeons Committee on Trauma's Firearm Strategy Team (FAST) Workgroup: Chicago Consensus I. *J Am Coll Surg*. 2019;228(2):198-206. https://doi.org/10.1016/j.jamcollsurg.2018.11.002
8. Barkin SL, Finch SA, Ip EH, et al. Is office-based counseling about media use, timeouts, and firearm storage effective? Results from a cluster-randomized, controlled trial. *Pediatrics*. 2008;122(1):e15-e25. https://doi.org/10.1542/peds.2007-2611
9. Albright TL, Burge SK. Improving firearm storage habits: impact of brief office counseling by family physicians. *J Am Board Fam Pract*. 2003;16(1):40-46. https://doi.org/10.3122/jabfm.16.1.40
10. Ranney M, Karb R, Ehrlich P, Bromwich K, Cunningham R, Beidas RS; FACTS Consortium. What are the long-term consequences of youth exposure to firearm injury, and how do we prevent them? A scoping review. *J Behav Med*. 2019;42(4):724-740. https://doi.org/10.1007/s10865-019-00035-2
11. Rowhani-Rahbar A, Zatzick D, Wang J, et al. Firearm-related hospitalization and risk for subsequent violent injury, death, or crime perpetration: a cohort study. *Ann Intern Med*. 2015;162(7):492-500. https://doi.org/10.7326/M14-2362
12. Barkin S, Duan N, Fink A, Brook RH, Gelberg L. The smoking gun: do clinicians follow guidelines on firearm safety counseling? *Arch Pediatr Adolesc Med*. 1998;152(8):749-756. https://doi.org/10.1001/archpedi.152.8.749
13. Stokes SC, McFadden NR, Salcedo ES, Beres AL. Firearm injuries in children: a missed opportunity for firearm safety education. *Inj Prev*. 2021;27(6):554-559. https://doi.org/10.1136/injuryprev-2020-044051
14. Madsen TE, Riese A, Choo EK, Ranney ML. Effects of a web-based educational module on pediatric emergency medicine physicians' knowledge, attitudes, and behaviors regarding youth violence. *West J Emerg Med*. 2014;15(5):615-622. https://doi.org/10.5811/westjem.2014.4.21365
15. McKay S, Bagg M, Patnaik A, et al. Addressing firearm safety counseling: integration of a multidisciplinary workshop in a pediatric residency program. *J Grad Med Educ*. 2020;12(5):591-597. https://doi.org/10.4300/JGME-D-19-00947.1
16. Clary C, Lambarth L, Kaushik R. Locked and (un)-loaded discussions: a pediatric resident safe firearm storage counseling curriculum. *MedEdPORTAL*. 2020;16:11028. https://doi.org/10.15766/mep_2374-8265.11028
17. Dingeldein L, Sheehan K, Krcmarik M, Dowd MD. Evaluation of a firearm injury prevention web-based curriculum. *Teach Learn Med*. 2012;24(4):327-333. https://doi.org/10.1080/10401334.2012.715254
18. Shafii T, Rivara FP, Wang J, Jurkovich GJ. Screening adolescent patients admitted to the trauma service for high-risk behaviors: who is responsible? *J Trauma*. 2009;67(6):1288-1292. https://doi.org/10.1097/TA.0b013e3181847e8e
19. Carter MB, Wesley G, Larson GM. Lecture versus standardized patient interaction in the surgical clerkship: a randomized prospective cross-over study. *Am J Surg*. 2006;191(2):262-267. https://doi.org/10.1016/j.amjsurg.2005.06.045
20. Park KY, Park HK, Hwang HS. Group randomized trial of teaching tobacco-cessation counseling to senior medical students: a peer role-play module versus a standardized patient module. *BMC Med Educ*. 2019;19:231. https://doi.org/10.1186/s12909-019-1668-x
21. Wintemute GJ, Betz ME, Ranney ML. Yes, you can: physicians, patients, and firearms. *Ann Intern Med*. 2016;165(3):205-213. https://doi.org/10.7326/M15-2905
22. Betz ME, Wintemute GJ. Physician counseling on firearm safety: a new kind of cultural competence. *JAMA*. 2015;314(5):449-450. https://doi.org/10.1001/jama.2015.7055
23. Solnick SJ, Hemenway D. Unintentional firearm deaths in the United States 2005–2015. *Inj Epidemiol*. 2019;6:42. https://doi.org/10.1186/s40621-019-0220-0
24. Fowler KA, Dahlberg LL, Haileyesus T, Annest JL. Firearm injuries in the United States. *Prev Med*. 2015;79:5-14. https://doi.org/10.1016/j.ypmed.2015.06.002
25. The BulletPoints Project: clinical tools for preventing firearm injury. BulletPoints Project. Accessed January 27, 2022. https://www.bulletpointsproject.org/
26. Kaufman DM. Applying educational theory in practice. *BMJ*. 2003;326(7382):213-216. https://doi.org/10.1136/bmj.326.7382.213
27. Solomon BS, Duggan AK, Webster D, Serwint JR. Pediatric residents' attitudes and behaviors related to counseling adolescents and their parents about firearm safety. *Arch Pediatr Adolesc Med*. 2002;156(8):769-775. https://doi.org/10.1001/archpedi.156.8.769
28. Hoops K, Crifasi C. Pediatric resident firearm-related anticipatory guidance: why are we still not talking about guns? *Prev Med*. 2019;124:29-32. https://doi.org/10.1016/j.ypmed.2019.04.020
29. Cohen LR, Runyan CW. Barriers to pediatric injury prevention counseling. *Inj Prev*. 1999;5(1):36-40. https://doi.org/10.1136/ip.5.1.36
30. Price JH, Thompson A, Khubchandani J, Wiblishauser M, Dowling J, Teeple K. Perceived roles of emergency department physicians regarding anticipatory guidance on firearm safety. *J Emerg Med*. 2013;44(5):1007-1016. https://doi.org/10.1016/j.jemermed.2012.11.010

Copyright © 2022 Stokes et al. This is an open-access publication distributed under the terms of the Creative Commons Attribution license.

31. Perry CD, Kenney GM. Differences in pediatric preventive care counseling by provider type. *Ambul Pediatr*. 2007;7(5):390-395. https://doi.org/10.1016/j.ambp.2007.06.001

32. Roszko PJD, Ameli J, Carter PM, Cunningham RM, Ranney ML. Clinician attitudes, screening practices, and interventions to reduce firearm-related injury. *Epidemiol Rev*. 2016;38(1):87-110. https://doi.org/10.1093/epirev/mxv005

33. Olson LM, Christoffel KK, O'Connor KG. Pediatricians' involvement in gun injury prevention. *Inj Prev*. 2007; 13(2):99-104. https://doi.org/10.1136/ip.2006.012401

Received: June 13, 2021
Accepted: January 12, 2022
Published: May 10, 2022

Copyright © 2022 Stokes et al. This is an open-access publication distributed under the terms of the Creative Commons Attribution license.
